# Genomic Diversity as a Key Conservation Criterion: Proof‐of‐Concept From Mammalian Whole‐Genome Resequencing Data

**DOI:** 10.1111/eva.70000

**Published:** 2024-09-10

**Authors:** Jong Yoon Jeon, Andrew N. Black, Erangi J. Heenkenda, Andrew J. Mularo, Gina F. Lamka, Safia Janjua, Anna Brüniche‐Olsen, John W. Bickham, Janna R. Willoughby, J. Andrew DeWoody

**Affiliations:** ^1^ Department of Forestry and Natural Resources Purdue University West Lafayette Indiana USA; ^2^ Western Association of Fish and Wildlife Agencies Boise Idaho USA; ^3^ Department of Biological Sciences Purdue University West Lafayette Indiana USA; ^4^ College of Forestry, Wildlife, and Environment Auburn University Auburn Alabama USA; ^5^ Center for Macroecology, Evolution and Climate, Globe Institute University of Copenhagen Copenhagen Denmark; ^6^ Department of Ecology and Conservation Biology Texas A&M University College Station Texas USA

**Keywords:** autozygosity, effective population size, evolutionary potential, genetic diversity, heterozygosity, sustainability, Watterson's theta

## Abstract

Many international, national, state, and local organizations prioritize the ranking of threatened and endangered species to help direct conservation efforts. For example, the International Union for Conservation of Nature (IUCN) assesses the Green Status of species and publishes the influential Red List of threatened species. Unfortunately, such conservation yardsticks do not explicitly consider genetic or genomic diversity (GD), even though GD is positively associated with contemporary evolutionary fitness, individual viability, and with future evolutionary potential. To test whether populations of genome sequences could help improve conservation assessments, we estimated GD metrics from 82 publicly available mammalian datasets and examined their statistical association with attributes related to conservation. We also considered intrinsic biological factors, including trophic level and body mass, that could impact GD and quantified their relative influences. Our results identify key population GD metrics that are both reflective and predictive of IUCN conservation categories. Specifically, our analyses revealed that Watterson's theta (the population mutation rate) and autozygosity (a product of inbreeding) are associated with the current Red List categorization, likely because demographic declines that lead to “listing” decisions also reduce levels of standing genetic variation. We argue that by virtue of this relationship, conservation organizations like IUCN could leverage emerging genome sequence data to help categorize Red List threat rankings (especially in otherwise data‐deficient species) and/or enhance Green Status assessments to establish a baseline for future population monitoring. Thus, our paper (1) outlines the theoretical and empirical justification for a new GD‐based assessment criterion, (2) provides a bioinformatic pipeline for estimating GD from population genomic data, and (3) suggests an analytical framework that can be used to measure baseline GD while providing quantitative GD context for consideration by conservation authorities.

## 1 Introduction

Global biodiversity is declining rapidly as humans continually modify natural habitats and expand our environmental footprint. Habitat reduction and fragmentation, overharvesting, invasive species, and other anthropogenic impacts routinely lead to population declines, reduced gene flow, and subsequent increases in inbreeding and genetic drift (Almeida‐Rocha et al. 2020; Schlaepfer et al. 2018). Collectively, these anthropogenic impacts lead to a loss of genetic/genomic diversity (GD) and a concomitant reduction in population fitness. The loss of GD and fitness can accelerate an extinction vortex (Blomqvist et al. 2010; Gilpin and Soulé 1986) and jeopardize the sustainability of a population or species because GD provides the evolutionary potential needed to adapt to a changing environment (DeWoody et al. 2021; England et al. 2003; Frankham 2005; Kardos et al. 2021). In this regard, the Convention on Biological Diversity (CBD) recently listed maintaining GD as an important goal in the Post‐2020 Global Biodiversity Framework with support from the International Union for Conservation of Nature (IUCN) (Hoban et al. 2020). As one of three components of biodiversity (along with species and ecosystem diversity), GD should be a central component of modern conservation policies.

As an international entity comprised largely of academic, government, and private members, IUCN strives to help protect nature by using the best available science to prioritize conservation efforts. For example, one of IUCN's major undertakings is the production of the “Green Status of Species” to help measure the recovery of populations and species which are subject to active conservation efforts (Akçakaya et al. 2018). A more ominous task is IUCN's production and regular updating of their “Red List,” which classifies species into one of nine categories (Extinct, Extinct in the Wild, Critically Endangered, Endangered, Vulnerable, Near Threatened, Least Concern, Data Deficient, and Not Evaluated). The Red List often influences national and state authorities in their official listing decision for species under their supervision. For example, the IUCN Red List is used at the international level by the Convention on International Trade in Endangered Species (CITES), CBD, and by the United Nations Sustainable Development Goals (SDGs). The IUCN Red List is also used at the national level by the National Institute of Biological Resources of South Korea and the U.S. Fish and Wildlife Service, and at the state or provincial level by the California Department of Fish and Wildlife and by the Indiana Department of Natural Resources (among many others). Ultimately, decisions made by IUCN regarding the Red List have long reverberated through the global conservation community and we expect future Green Status assessments to be similarly influential.

As it now stands, Red List assignments do not explicitly consider GD despite numerous calls to do so (Garner, Hoban, and Luikart 2020; Laikre et al. 2020; van Oosterhout 2020; Willoughby et al. 2015). Similarly, IUCN's Green Status metrics do not currently include GD although recent papers argue that it should (Jackson et al. 2022; van Oosterhout et al. 2022; van Oosterhout 2024). This is unfortunate because GD is an important component of population viability (Kardos et al. 2021; Reed and Frankham 2003), and in many instances GD can provide insights into rare or elusive species whose population attributes are otherwise difficult to address (Brüniche‐Olsen, Westerman, et al. 2018; Khan et al. 2021). For example, Rice's whale (*Balaenoptera ricei*) is a newly described species of baleen whale endemic to the Gulf of Mexico (Rosel et al. 2021). Baleen whales are notoriously difficult to study at sea, but empirical GD estimates from only a few individuals (e.g., sourced from beached whales or noninvasively collected DNA) could provide both baseline population genomic data for future monitoring as well as critical demographic context for conservation plans.

Previous studies based on microsatellite genetic markers have suggested that threshold levels of GD can be used to help delimit conservation categories. For instance, Willoughby et al. (2015) proposed a conceptual framework that—based strictly on GD estimates from related species and recognizing that GD is but one component of population viability—designates IUCN conservation categories based on the estimated time (in generations) that a species or population is predicted to lose more GD than 75% of its taxonomic relatives. The conceptual framework of Willoughby et al. (2015) was proposed at the twilight of the microsatellite era. Here, we extend it into the dawning population genomic era. Although previous genomic studies have explored the relationship between GD and conservation status (e.g., Brüniche‐Olsen, Kellner, et al. 2018; Genereux et al. 2020; Wilder et al. 2023), they used only a single genome per species. Kardos et al. (2021) insisted that population‐level data are necessary to accurately reflect genetic variation because a single individual may not properly represent the species as a whole, and we agree. Genome resequencing datasets from natural populations are on the rise (DeWoody et al. 2022) and they provide rich new opportunities for conservation‐informative GD metrics to be molded into one or more formal conservation criteria.

The IUCN Red List makes categorical assignments for each species they evaluate according to five criteria: (1) population size reduction; (2) geographic range; (3) small population size and decline; (4) very small or restricted population; and (5) associated quantitative analyses of population viability. In contrast, Green Status assessments evaluates the recovery status of species based on a “Green Score” that ranges from 0% to 100% where 100% represents fully recovered (i.e., higher scores indicate better recoveries).

In this paper, we first assessed whether IUCN yardsticks effectively reflect contemporary mammalian GD. We reasoned that if so, Red List of Threatened species (Critically Endangered, Endangered, and Vulnerable) should exhibit lower levels of GD than Non‐Threatened species (Near Threatened and Least Concern) due to inbreeding, genetic drift, and reduced gene flow. If not, this would indicate that the Red List evaluation criteria insufficiently capture a key aspect of biological diversity (i.e., GD; see van Oosterhout 2024). We then thought about how GD could be utilized in Green Status assessments, then developed an explicit algorithm for doing so. We focused primarily on Watterson's theta (*θ*_W_), the number of segregating sites in a gene pool (i.e., *θ*_W_ = 4*N*_e_ *μ* for diploids under mutation‐drift equilibrium; Watterson 1975). Watterson's theta can serve simultaneously as an effective metric of GD and as a proxy for effective population size (*N*_e_), both useful additions to IUCN assessments in part because of the inherent difficulties in consistently estimating *N*_e_ across studies (Waples 2024a). Unlike some lagging indicators of GD such as nucleotide diversity (which may reflect more ancient demographic events such as bottlenecks or expansions), *θ*_W_ is a leading indicator of GD because it depends more on contemporary *N*_e_ (Tajima 1989b; Brüniche‐Olsen et al. 2021).

Unfortunately, a simple fixed GD threshold for conservation (e.g., mean *θ*_W_ < 0.002 = Endangered) would be misleading because of the inherent variation in GD observed among taxa. Species vary in key biological attributes such as body sizes, generation times, and metabolic rates that are known to affect GD (Bromham 2024; Ellegren and Galtier 2016; Romiguier et al. 2014). Thus, we examined associations among fundamental biological characteristics, such as trophic level and body mass, with GD to account for major biological factors that might otherwise confound the relationship between GD and Red List status. We did so using Class Mammalia as an example because many flagship species of conservation interest (e.g., pandas, tigers, and whales) are mammals. Furthermore, mammalian data are sufficiently dense in both the IUCN Red List and in sequence repositories to allow for robust analyses of our GD framework. Finally, we provide explicit suggestions for how authorities might improve the Red List and Green Status assessments—and hopefully improve subsequent conservation outcomes—by incorporating GD.

## 2 Materials and Methods

The overall workflow of this study is illustrated in Appendix A1. In addition to *θ*_W_, we also evaluated four other commonly utilized population genomic metrics that each has a strong theoretical justification for being conservation‐informative: (1) mean nucleotide diversity (𝜋); (2) mean observed genome‐wide heterozygosity (*H*); (3) Tajima's *D* (*D*); and (4) the extent of autozygosity as measured by runs of homozygosity (ROH). Nucleotide diversity, *𝜋*, conveys the average number of nucleotide differences per site between all pairs of sequences in a population. Heterozygosity, *H*, measures the proportion of heterozygous sites considered in a given sample (Nei 1978). At the population level, mean *H* is averaged across estimates from individual genomes. Tajima's *D* is the difference between *𝜋* and *θ*_W_, divided by its variance under mutation‐drift equilibrium (Tajima 1989a). Tajima's *D* can be used to identify signatures of selection on individual loci, but demographic trends can also be detected when it is measured across the genome: *D* < 0 indicates population growth after a bottleneck, *D* = 0 indicates population stability, and *D* > 0 indicates a sudden population decline. Finally, ROHs describe the proportion of contiguous homozygous regions along the genome and can used to directly estimate both the extent and timing of inbreeding (and indirectly, the level of inbreeding depression due to associated reductions in fitness) (Ceballos et al. 2018). We calculated two ROH estimators, namely *F*_ROH>100kb_ (the fraction of ROH longer than 100 kb, *F*100kb; representing the cumulative inbreeding level) and *F*_ROH>1Mb_ (the fraction of ROH longer than 1 Mb, *F*1Mb; representing the recent inbreeding level).

### 2.1 Data Collection

We collected four types of data from public databases: (1) reference genomes; (2) population‐level whole genome resequencing (WGR) reads for the inference of GD metrics; (3) IUCN Red List information; and (4) ecological characteristics (trophic level, body mass, and habitat breadth) for statistical tests of association with the GD metrics. Because formal taxonomic designations (e.g., subspecies) do not always perfectly correspond with conservation units, we used subspecies or regional population level data from the Red List whenever available because conservation status can vary among demographically independent populations within the same species.

We searched all the available reference genomes of mammalian species (as of December 2021) from National Center for Biotechnology Information (NCBI) and collected assembly identifiers (e.g., accession number and assembly name) required for our bioinformatic pipeline. We also collected additional information on the assembly level (i.e., contig, scaffold, or chromosome), contig N50, scaffold N50, and assembly size for downstream analyses (Dataset S1). Species with a reference genome were further searched and population‐level WGR data were retrieved from NCBI's Sequence Read Archive (SRA). We use population‐level data when: (1) “WGS” type data were available; (2) the data were comprised of paired‐end reads; (3) the data were sequenced on Illumina platforms, such as Genome Analyzer, Hiseq, Novaseq, or Nextseq platforms; and lastly (4) a minimum of two different individuals from the same wild population were available. We followed the data author's population designation and limited the maximum number of individuals to 25 for computational tractability. The largest population was selected when multiple populations were available. We recorded the Illumina sequencing chemistry used (i.e., two‐channel or four‐channel; De‐Kayne et al. 2021) and the number of individuals for downstream use (Dataset S1, https://github.com/jyj5558/theta).

For each species evaluated, we used the IUCN Red List to record conservation category (i.e., CR—Critically Endangered, EN—Endangered, VU—Vulnerable, NT—Near Threatened, LC—Least Concern), population trend (i.e., decreasing, stable, or increasing), and extent of geographic range. We imported the shape file of the species geographic range to ArcGIS Pro 2.9.0 (Esri 2021) and calculated the total habitat ranges except for “Extinct” and “Possibly Extinct” species. The shape files were clipped to match with the collection site of “subspecies” or “subpopulation,” as defined by the Red List, whenever apparent and applicable based on the associated metadata.

### 2.2 Bioinformatic Pipeline

We downloaded each species' reference genome assembly, sorted them by length using BBMap v37.93 (https://sourceforge.net/projects/bbmap/) and indexed each using samtools v1.8 (Li et al. 2009). Short scaffolds <100 kb, including mitochondrial sequences, were also removed. Repeat files were downloaded from the assembly file if readily available or were created by running RepeatMasker v4.0.7 (Smit, Hubley, and Green 2015) with “quick” option (−qq) using the mammalian repeat database.

For each species, WGR SRA files (fastq format) were downloaded using the sra‐toolkit v2.11.0 (https://github.com/ncbi/sra‐tools). We employed FastQC v0.12.1 to check the raw quality of downloaded fastq files and TrimGalore v0.6.10 (https://www.bioinformatics.babraham.ac.uk/projects/trim_galore/) to cull adapter sequences (using “very stringent” setting), low‐quality ends (<20 nt) or reads of short length (<30 nt), and read pairs of short length (<30 nt). Sequence quality was checked again after filtering and samples where <80% of reads passed quality filters were removed from downstream analyses.

Quality‐filtered SRA reads from each species were mapped onto the respective reference assembly using bwa‐mem v0.7.17 (Li and Durbin 2009) after creating a reference genome dictionary with Picard tools v2.9.0 (http://broadinstitute.github.io/picard/). To improve read mapping quality, we locally realigned reads using GATK v3.8.1's “RealignerTargetCreator” and “IndelRealigner” tools (van der Auwera and O'Connor 2020). We used samtools to estimate summary statistics (mapping rate, depth, and breadth of coverage) from the resultant bam files and low quality data (<80% mapping rate, <1x depth, or <80% breadth) were removed. We estimated mappability using GenMap v1.3.0 (Pockrandt et al. 2020) with 100‐bp k‐mer setting allowing two mismatches. Sites with low mappability (<1) were not considered. Non‐repeat regions were identified from the length‐filtered reference genome using bedtools v2.29.0 complement (Quinlan and Hall 2010). Intersecting regions among the non‐repeat regions, regions of mappability = 1, and scaffolds longer than 100 kb were identified using bedtools and used in the downstream analyses.

We estimated GD metrics using ANGSD v0.94.0 (Korneliussen, Albrechtsen, and Nielsen 2014) and bcftools v1.17 (Danecek et al. 2021). We applied conservative filters in ANGSD, including removing low quality reads and ambiguously mapped reads. We estimated genotype likelihoods with GATK and maximum likelihood estimates of the folded site frequency spectrum were obtained using the realSFS tool. We estimated *𝜋*, *θ*_W_, and *D* applying a sliding window approach with non‐overlapping 50 kb windows. Estimates of *𝜋* and *θ*_W_ were divided by the effective number of sites to represent genomic proportions. Individual genome‐wide *H* was estimated using a similar process and averaged to provide a population‐level mean *H* estimate for each species. To estimate the ROH burden, a bcf file was generated from quality filtered bam files using ANGSD. Subsequently, bcftools/roh (Narasimhan et al. 2016) was employed to apply the hidden Markov model to identify individual ROHs. The fraction of ROHs in individual genomes (*F*_ROH_) were averaged to obtain a population‐level estimate per species using an in‐house python script.

### 2.3 Statistical Analysis

Descriptive statistics (mean and standard deviation) and the distribution of GD metrics were summarized and plotted by both IUCN categories and taxonomic Orders. Before the main analyses described below, we partitioned the full dataset (Dataset S1) into two subsets. The first subset, the “IUCN dataset” (Dataset S2), included all the species having their own categorical Red List assessment but excluded those listed as “Data Deficient.” The second subset, the “EcoEvo dataset” (Dataset S3), included all the species with eco‐evolutionary traits available in the COMBINE database (Soria et al. 2021). Uncorrelated GD metrics were then individually tested against IUCN categories to determine if there was a significant difference between mean GD values across IUCN categories. We used both “full” categories (CR, EN, VU, NT, and LC) as well as “binary” categories consisting of Threatened (=CR + EN + VU) or Non‐Threatened (=NT + LC). We considered technical factors as well (e.g., sequence read depth) and controlled for them in statistical models (Technical Dimensions; Dim.1–Dim.4) as described in Appendix A2. To account for phylogenetic signal (*𝜆*), we ran Phylogenetic Generalized Least Squares models (PGLS; GD ~ IUCN category + Dim.1 + Dim.2 + Dim.3 + Dim.4–1) using the R package “caper” (Orme et al. 2018) with the maximum likelihood method. The significance of individual independent variables was further examined in each model and effect sizes (partial omega‐squared) were determined for the independent variables of interest using R package “sjstats” (Lüdecke 2022). Comparisons among significant models were assessed using Akaike information criterion (AIC) values.

The mammalian phylogenetic tree used in the models was derived from VertLife (Upham, Esselstyn, and Jetz 2019) by sampling 1000 trees from the “Mammals birth‐death node‐dated completed trees” distribution (Upham, Esselstyn, and Jetz 2019). The “averageTree” function with default settings was applied using the R package “phytools” (Revell 2012) to obtain a consensus tree from the 1000 trees, which was then rooted with *Sarcophilus harrisii* as an outgroup (Damas et al. 2022) using the “root” function in R package “ape” (Paradis and Schliep 2019). Additional tips for each subspecies were manually added to the tree as a sister taxon of its closest relative using the “AddTip” function in the R package “TreeTools” (Smith 2019). Two Red List assessment criteria, “population trend” and “geographic range,” were compared in place of the IUCN category with the same procedure above.

We used ordinal regression tests to examine the explanatory power of GD metrics that were significantly correlated with IUCN categorization after accounting for phylogenetic non‐independence. Each model consisted of IUCN full categories or binary categories (i.e., threatened vs. non‐threatened) as dependent variables, and one of the significant GD metrics as an independent variable. IUCN categories were treated as pseudo‐continuous following (Graber 2013).

For combinations of GD metrics and IUCN categorizations that covaried according to phylogenetic history in both PGLS tests, we ran multi‐response phylogenetic mixed modeling (MR‐PMM) using the R packages “MCMCglmm” (Hadfield 2010). In these tests, both a GD metric and IUCN categorization were treated as response variables to distinguish and estimate phylogenetic and independent covariances (Halliwell, Yates, and Holland 2023; Westoby et al. 2023). For each test, various priors were tested with Markov chain Monte Carlo (MCMC) sampling to obtain the best model convergence, then one set of priors that achieved successful convergence was fixed to the test. Models were run for 11,000,000 iterations including a 1000,000 burn‐in period and sampling every 1000 runs. Model convergence was assessed by effective sample sizes and trace plots. We then estimated the link‐scale independent (i.e., non‐phylogenetic) correlations between GD metrics and IUCN categorization. Detailed R code is available at https://github.com/jyj5558/theta.

We considered several machine learning classifiers (i.e., random forest, k‐nearest neighbors, and support vector machine) that are applicable to small datasets using the “scikit‐learn” package (Osisanwo et al. 2017). Only GD metrics identified as significant in linear models were included as predictors, and IUCN binary categories were included as responses. For the random forest and linear support vector machine classifiers, we used predictors simultaneously and compared their feature importance. For k‐nearest neighbors and non‐linear support vector machine classifiers, we used predictors individually and compared their model accuracy. Predictors were standardized in k‐nearest neighbors and support vector machine models. We used 70% of the data for model training and 30% for model testing. After hyperparameter tuning by a wide range of randomized grid searches and/or a finer parameter grid search for each machine learning classifier, the final models were set according to the best estimator.

We tested associations between IUCN categories and (a) population trend or (b) geographic range estimates, two criteria currently used to help determine IUCN status. We did so to provide perspective on the signal (or lack thereof) of individual criteria contained in GD metrics.

To strengthen our conservation‐oriented analyses by accounting for potential confounding factors, the distribution of all the GD metrics, conservation criteria and eco‐evolutionary factors across species was displayed on a multi factor analysis (MFA) plot using R packages “FactoMineR” (Lê, Josse, and Husson 2008) with the first two dimensions. We also leveraged the dataset to evaluate evolutionary factors by comparing GD against key eco‐evolutionary factors that could drive levels of standing GD, including trophic level, habitat breadth, and body mass. See Appendix A3 for details.

## 3 Results

### 3.1 Genetic Diversity Across Red List Categories and Across Species

Among 613 species and subspecies with reference genome assemblies available from NCBI at the time of our search in 2022, 98 species had population genomic whole‐genome resequencing (WGR) datasets that met our inclusion criteria. Sixteen species were subsequently dropped during the bioinformatic data analysis due to unsatisfied thresholds (e.g., low mapping rates, depths, and/or breadth) or an inconsistent data type (e.g., pooled‐sequencing). Ultimately, this resulted in 82 species in our final WGR dataset (Dataset S1). Our “IUCN” (Dataset S2) and “EcoEvo” (Dataset S3) datasets had 72 and 63 species, respectively, after reconciling taxonomy and pruning for phylogenetic pseudoreplication.

**FIGURE 1:**
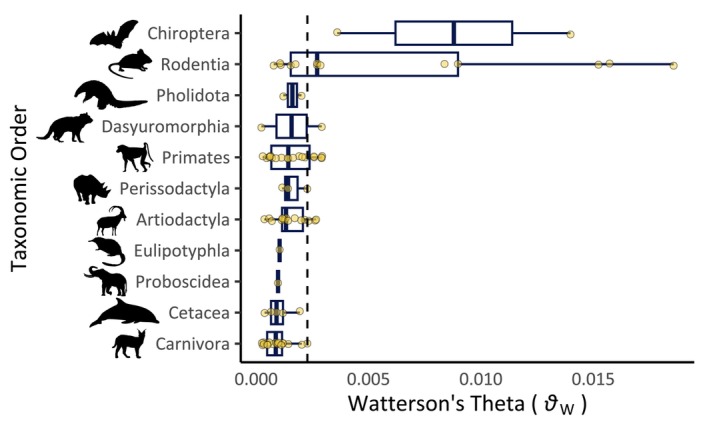
A box plot of Watterson's theta by taxonomic Order. Taxonomic Orders are arranged by descending median value of diversity. Dashed line indicates the overall mean value. Silhouette images of animals are adapted from PhyloPic (https://www.phylopic.org/).

**FIGURE 2:**
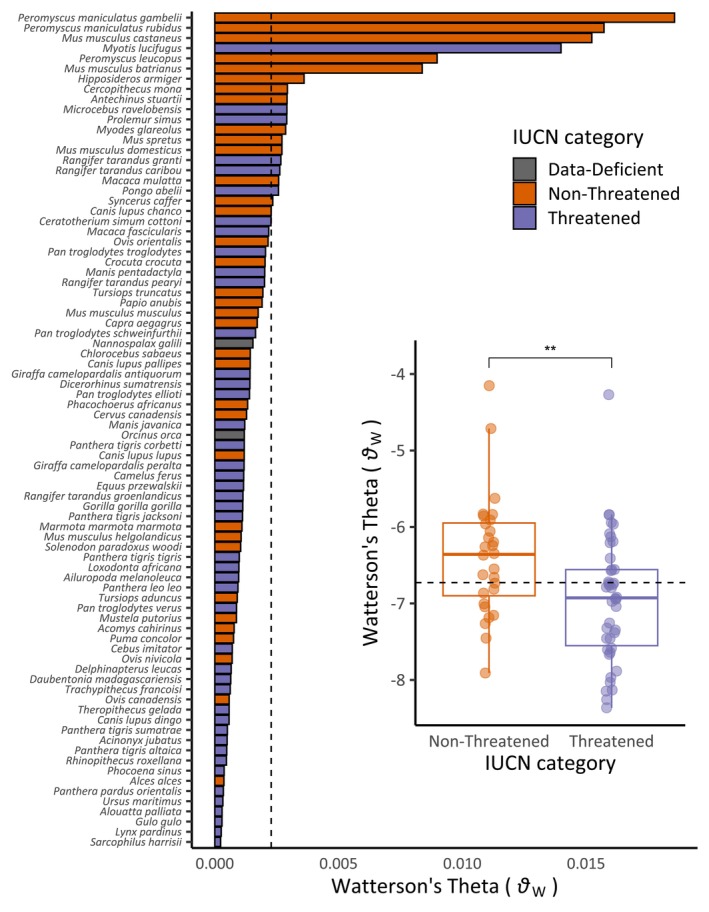
A bar plot of Watterson's theta (*θ*_W_) by species. Species are arranged by descending value of Watterson's theta and colored by IUCN Threatened/Non‐Threatened categories, plus “Data‐Deficient.” Dashed line indicates the overall mean value. Species names according to NCBI are shown on the *y*‐axis. The inset shows a box plot of log‐transformed Watterson's theta against IUCN categories. The box represents the range between the first and the third quartile (interquartile range, IQR) with the median line inside. The whiskers above and below the box represent the largest and smallest values within 1.5 IQR, respectively. Non‐Threatened category is compared to Threatened category using a Wilcoxon test and the significance is shown (**p < 0.01). Dashed line indicates the overall mean value.

Descriptive statistics for each GD metric are summarized in Table 1 and Table S1 (see also Figure 1 and Figures S1–S14). Out of 82 species, 21 species did not yield *F*_ROH>1Mb_ (*F*1Mb) estimates due to low contiguity of their reference genome. Nucleotide diversity (*𝜋*) and genome‐wide heterozygosity (*H*) values were strongly correlated (*r* > 0.9) with Watterson's theta (*θ*_W_). Thus, while we interpreted results mainly based on *θ*_W_ as it is more sensitive to population decline, we employed *H* for practical assessment criterion below (see Section 4.3). In general, Non‐Threatened species have higher GD than Threatened species. Individual and categorical *θ*_W_ was effectively twice as high in Non‐Threatened species compared to Threatened species (Table 1 and Figure 2), and *F*1Mb (the metric of recent inbreeding) was doubled in Threatened species (Table 1 and Figure S14).

**TABLE 1:** Genomic diversity metrics grouped by IUCN full categories.

IUCN category	Spp. no.	Mean (*θ*_W_)	SD (*θ*_W_)	Mean (*H*)	SD (*H*)	Mean (*π*)	SD (*π*)
DD	2	0.00137	0.00025	0.00152	0.00031	0.00151	0.00042
LC	30	0.00360	0.00481	0.00319	0.00344	0.00331	0.00365
NT	5	0.00201	0.00076	0.00255	0.00094	0.00237	0.00085
VU	15	0.00119	0.00091	0.00121	0.00089	0.00131	0.00093
EN	15	0.00187	0.00341	0.00176	0.00280	0.00198	0.00354
CR	15	0.00133	0.00079	0.00145	0.00081	0.00140	0.00081
Non‐Threatened	35	0.00338	0.00449	0.00310	0.00320	0.00317	0.00340
Threatened	45	0.00147	0.00206	0.00148	0.00174	0.00157	0.00213

IUCN category	Spp. no.	Mean (*D*)	SD (*D*)	Mean (*F*100kb)	SD (*F*100kb)	Mean (*F*1Mb)	SD (*F*1Mb)
DD	2	0.36770	0.42422	0.07753	0.10954	0.01300	NA
LC	30	0.17316	0.70542	0.08145	0.08283	0.02607	0.04050
NT	5	0.65424	0.47784	0.17202	0.10911	0.01980	0.02357
VU	15	0.69864	0.45017	0.11886	0.08329	0.06752	0.07027
EN	15	0.45159	0.55982	0.12005	0.09415	0.04228	0.04155
CR	15	0.26068	0.88502	0.09922	0.06770	0.02603	0.04628
Non‐Threatened	35	0.24189	0.69316	0.09439	0.09103	0.02477	0.03724
Threatened	45	0.47030	0.66805	0.11271	0.08111	0.04407	0.05430

Abbreviations: *D*, Tajima's *D*; *F*100kb, *F*_ROH>100kb_; *F*1Mb, *F*_ROH>1Mb_; *H*, observed genome‐wide heterozygosity; NA, not applicable; Spp. no., the number of species; *θ*_W_, Watterson's theta; *𝜋*, nucleotide diversity.

Bats and rodents (Order Chiroptera and Rodentia, respectively) had the highest mean *θ*_W_, *𝜋*, and *H* values, almost triple the next most genetically diverse Order (Artiodactyla; even‐toed ungulates). Carnivores and large mammals (Orders Proboscidea and Cetacea) are at the other end of the GD distribution. Mean *D* was lowest among Proboscidea (elephants), Cetacea, Rodentia and highest among Eulipotyphla (hedgehogs and relatives), Pholidota (pangolins), and Carnivora. Chiroptera had the lowest mean *F*100kb while Eulipotyphla, Carnivora, and Primates had the highest *F*100kb. Dasyuromorphia, Pholidota, and Proboscidea had the lowest mean *F*1Mb while Carnivora, Primates, Rodentia had the highest.

### 3.2 Statistical Associations Between GD and the IUCN Red List

Overall, our analyses indicate that two GD metrics—*θ*_W_ and *F*100kb—were significantly associated with IUCN conservation categories (detailed model results are presented in Table S2 and Figures S15–S17). The main phylogenetic generalized least squares (PGLS) model between IUCN full categories (Least Concern—LC, Near Threatened—NT, Vulnerable—VU, Endangered—EN, Critically Endangered—CR) and *θ*_W_ was significant (significant partial *ω* ^2^ = 0.099). There was significant phylogenetic signal (*𝜆*) in the model (*𝜆* = 0.961; 95% CI = 0.883–0.988), implying there was phylogenetic non‐independence in the data which was accounted for in the model. The secondary PGLS model of *θ*_W_ against IUCN binary categories of Threatened and Non‐Threatened (LC + NT + VU = Non‐Threatened; EN + CR = Threatened) was also significant with *𝜆* = 0.951 (significant partial *ω* ^2^ = 0.106). The PGLS models also revealed a significant relationship between the ROH burden (as measured by *F*100kb) and IUCN full categories (significant partial *ω* ^2^ = 0.106) and between the ROH burden and binary IUCN categories (non‐significant partial *ω* ^2^ = 0.001). None of the PGLS models revealed significant associations between *D* or *F*1Mb and IUCN category (full or binary). Among significant models, the *F*100kb with IUCN binary categories model was best, only slightly better than the model with IUCN full categories, followed by *θ*_W_ (Table S3). See Appendix A4 for the results of models which used individual Red List assessment criteria (i.e., “population trend” and “geographic range”) in place of the IUCN categories.

Our analyses indicate that *θ*_W_ and *F*100kb were significantly associated with conservation status. Thus, we used either *θ*_W_ or *F*100kb as independent variables in the PGLS for phylogenetic ordinal regression against IUCN categorization. The model with *θ*_W_ as the independent variable was significant when IUCN binary category was the dependent variable (significant partial *ω* ^2^ = 0.121; Table S2). Regardless of whether the dependent variable was the IUCN full category or binary category, *θ*_W_ was always significant as an individual factor whereas *F*100kb was never a significant predictor of IUCN category (whether full or binary; Table S2).

The results of MR‐PMM indicated that all models converged well with effective sample sizes reaching nearly 100% of the sampled chains for each estimated model parameter (Figures S18–S21). Both full and binary IUCN categories had a strong a phylogenetic signal (Table S4), with *θ*_W_ and *F*100kb both correlating with the IUCN categories after controlling for phylogenetic non‐independence, but *θ*_W_ was more strongly correlated. Among machine learning classifiers, *θ*_W_ was generally a better predictor of IUCN categories than *F*100kb. The K‐nearest neighbors approach with *θ*_W_ as a predictor of IUCN binary categorization showed the highest accuracy, followed by the linear support vector machine approach with *θ*_W_ and *F*100kb both as predictors and non‐linear support vector machine with *θ*_W_ as a predictor. However, the model accuracy (≤0.6) and importance of predictor (≤0.6) were relatively low for both *θ*_W_ and *F*100kb. The fine‐tuned hyperparameters and results for each of the machine learning classifiers are presented in Table S5.

## 4 Discussion

The relationships among GD, *N*_e_, and fitness have been thoroughly reviewed and summarized by previous studies (James and Eyre‐Walker 2020; Mitton 1994; Nevo, Beiles, and Ben‐Shlomo 1984). These and other studies indicate that GD, as estimated by *θ*_W_ or related measures such as individual heterozygosity, is a critical component not only of contemporary fitness but also of future evolutionary potential. The idea that GD can serve as an indicator of future evolutionary potential should not be overlooked considering the global environmental challenges facing natural populations today (but see Section 4.5). Furthermore, genome resequencing data offer remarkably high information content per individual (e.g., estimates of GD such as mean *θ*_W_ or *F*100kb). This means that sampling the genomes of only a few individuals can provide key insights into population biology, and this could be especially important in the case of rare and/or secretive species whose populations are difficult to survey using conventional means.

A reduction in GD, with its concomitant loss of fitness and increased probability of extinction (Flight 2010; Frankham 2005), is expected to result from demographic events like population bottlenecks, population subdivision, and founder events that reduce population sizes. Neutral GD is determined by the product of the generational mutation rate and the effective population size (*N*_e_), and thus GD is determined in part by the census size of the population (James and Eyre‐Walker 2020; Leffler et al. 2012). Moreover, and not surprisingly, population census size is positively correlated with geographic range size. According to conservation theory, small, threatened populations tend to have lower GD than large, broadly distributed populations which are typically not threatened (Frankham 1996).

Our analyses of empirical data bear out those theoretical predictions (Figure 2). We analyzed population genomic data from 82 species belonging to 11 Orders of mammals among various IUCN conservation categories. For each species, we calculated GD metrics and tested for significant associations between these metrics and various biological parameters, such as geographic distribution or body size, that might impact diversity. The overarching goal of the research was to determine the relationship between population‐level GD metrics and IUCN conservation categories while simultaneously identifying key intrinsic drivers of mammalian GD, which we address first.

### 4.1 Description of Mammalian Genomic Diversity

Our results are consistent with a long history of empirical genetic studies dating to the 1960s when protein electrophoresis was first used to measure GD in natural populations of mammals. For example, Figure 2 indicates that the four species with the highest *θ*_W_ values (i.e., the number of variable nucleotide sites) are *Peromyscus maniculatus* (deer mouse, including two subspecies), *Mus musculus castaneus* (southeastern Asian house mouse), and *Myotis lucifugus* (little brown bat) followed by *Peromyscus leucopus* (white‐footed mouse). Nevo, Beiles, and Ben‐Shlomo (1984) compiled an allozyme dataset of GD metrics (e.g., *H*) from 1111 species of animals and plants including 184 species of mammals. Their dataset was comprised of GD estimates from only a few dozen allozyme markers per species, and they examined only a few of the same species that we did. However, there are some remarkable similarities between Nevo, Beiles, and Ben‐Shlomo (1984) and our current study. Nevo, Beiles, and Ben‐Shlomo (1984) found only 13 species (not including humans and domestic cat) that had values of *H* > 0.09 out of 184 species of mammals. Among them were *P. maniculatus* and two species of bats of the genus *Myotis*. The taxonomic overlap in high GD species between Nevo, Beiles, and Ben‐Shlomo (1984) and our study is reassuring. We also found a strong correlation (*r* = 0.81) between our heterozygosity estimates and Nevo, Beiles, and Ben‐Shlomo's (1984) estimates among the 18 species included in both studies. These findings bolster our confidence that evolutionary genetics theory is buttressed by existing, publicly available genomic datasets that can be readily exploited by interested conservationists.

Taxonomic Order is the taxonomic level in which member species share a broad suite of morphological, physiological, genetic, and ecological characteristics; species of different Orders can easily be distinguished by many conservationists. If we just consider the four most speciose Orders, Rodentia had the highest mean value of *θ*_W_ = 0.00626 and Carnivora had the lowest mean value *θ*_W_ = 0.00090. This is not unexpected given that small herbivores generally have much larger population sizes and nucleotide substitution rates than do carnivores (Zhang et al. 2021). Conversely, Carnivores had the highest mean *F*1Mb = 0.06209 and rodents had the second lowest mean *F*1Mb = 0.02441. Again, this is consistent with their population biology in which rodents (a group of animals with small bodies, short lifespans, and high fecundity) are expected to have higher effective mutation rates and larger population sizes, thus higher GD, than carnivores (animals with larger bodies, longer lifespans, and lower fecundity), where there is generally far more opportunity for inbreeding in isolated populations (De Kort et al. 2021; Romiguier et al. 2014). Primates have relatively high inbreeding with *F*1Mb = 0.05569. This is perhaps a reflection of a high degree of social structuring, small census population sizes, and slower rates of molecular evolution in primates (Zhang et al. 2021).

### 4.2 Genomic Diversity and Red List Status

We found that key population GD metrics are generally reflective and predictive of IUCN conservation categories that presumably reflect extinction threat status. The effect sizes of the independent GD metrics (partial *ω* ^2^ = 0.099–0.121) indicate they explained a modest to large proportion of the variance in the response variable (Field 2013). This supports the idea that GD is indirectly reflected by the current Red List assessment methodology. Our results also indicate that Threatened species or populations have reduced GD compared to those with Non‐Threatened status. We found that *θ*_W_ (and its correlates, *𝜋* and *H*, which are both measures of genomic variation based on polymorphic nucleotide sites) was the best conservation metric, followed by *F*100kb (a measure of autozygosity that is reflective of inbreeding). One individual Red List criteria, “geographic range,” also reflected GD. Geographic range was inversely proportional to longer fraction of ROH (Figure A5), another reasonable result in that habitat contraction can result in elevated levels of inbreeding relative to random mating (Nonaka et al. 2019).

The correlation between GD and Red List status has been tested before (Brüniche‐Olsen, Kellner, et al. 2018; Brüniche‐Olsen et al. 2021; Garner, Hoban, and Luikart 2020; Nabholz et al. 2008; Willoughby et al. 2015) but mostly with mitochondrial sequences, microsatellite marker data, or a single genome sequence. There has been no scientific consensus on whether the Red List indirectly captures GD. Recently, Schmidt et al. (2023) performed a meta‐analysis of studies that used different markers and corroborated Willoughby et al. (2015), who found that GD is modestly predictive of Red List status. Our results are consistent with this interpretation. Several authors (Garner, Hoban, and Luikart 2020; Schmidt et al. 2023; Willoughby et al. 2015) have suggested the loss of GD over time would be even more valuable than snapshot values of GD in conservation assessments. In the next section, we extend this line of reasoning by detailing an approach for including GD as an explicit criterion in future conservation assessments.

### 4.3 An Explicit Genetic Criterion for Conservation Assessments

Over 30 years ago, Mace and Lande (1991) originally suggested an assessment criterion based on *N*_e_ in Version 1.0 of the Red List Categories and Criteria, but the most recent iteration of these Criteria (Version 3.1) still do not embrace *N*_e_ despite recent pleas to include genetic considerations in status determinations (e.g., Garner, Hoban, and Luikart 2020; Laikre 2010; Willoughby et al. 2015). Furthermore, *N*_e_ is the indicator proposed by the Global Biodiversity Framework (Hoban et al. 2020). Effective population size is notoriously difficult to estimate, but is a primary determinant of GD (e.g., Watterson 1975; equation 3 in Nei and Takahada 1993). Thus, we suggest that an additional criterion that considers GD and *N*_e_ would help further inform conservation assessments. We think an additional criterion could be useful for all species where GD data are available, but especially for species that might otherwise be deemed “Data Deficient” (i.e., limited data available to assess conservation status based on traditional criteria).

Our proposal for an explicit GD criterion for status assessments is based on the mean loss of heterozygosity over time (Crow and Kimura 1970). We chose *H* in large part because (1) our results indicate that since *H* is correlated with *θ*_W_, *H* is both a good indicator and good predictor of existing IUCN categories because the number of variable sites across a genome (the *θ*_W_ we used) is equal to *H* when calculated from a single individual; and (2) it has a solid theoretical foundation based on Crow and Kimura's equation. To accommodate a time frame (100 years) relevant for conservation programs and to align with Green Status assessments, we modified the original equation such that:

$$H_{T}=H_{O}{\left(1-\frac{1}{2N_{e}}\right)}^{T}$$

where *N*_e_ is the effective population size, *H*_O_ is observed heterozygosity, *H*_T_ is heterozygosity at time *T* where *T* is the number of generations in 100 years (e.g., *T* is 100 for most insects or annual plants, *T* is 50 for antelope with 2‐year generation times, and *T* is 5 for whales with 20‐year generation times). Furthermore, *H* has previously been proposed as a key genetic criterion for conservation efforts (Allendorf and Ryman 2002; Willoughby et al. 2015) and *H* can be accurately estimated from only a few whole genome sequences (Gorman and Renzi 1979; Nei and Roychoudhury 1974), an important consideration with respect to Threatened populations or species. Finally, and importantly, the concept of heterozygosity is well understood by most biologists.

**FIGURE 3:**
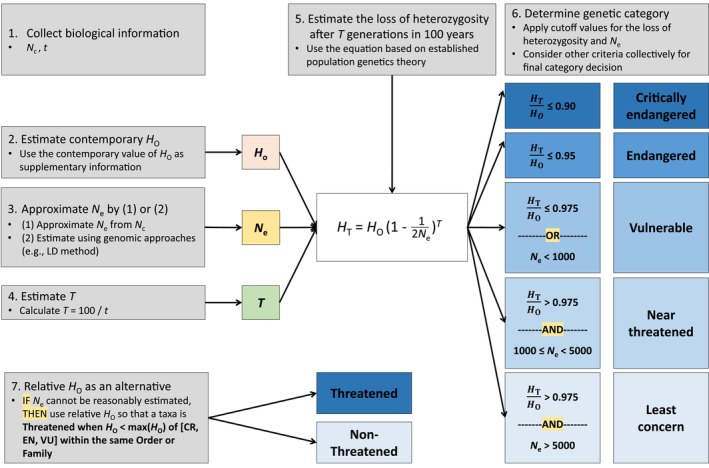
A practical outline of how genetic or genomic diversity (GD) could be explicitly used to help determine formal conservation status. The loss of heterozygosity after 100 years can be predicted and used to determine conservation status according to specific thresholds or by comparison to *H*_O_ estimates of related taxa. For example, even when *H*_T_/*H*_O_ > 0.975, if *N*_e_ < 1000 then the species or population would warrant categorization as VU as gauged exclusively on its GD. If *N*_e_ cannot be reasonably estimated (e.g., because *N*_c_ cannot be estimated), then a Threatened designation would be warranted when *H*_O_ < maximum *H*_O_ of species in “Threatened” genetic categories (i.e. VU, EN, or CR as determined by their *N*_e_ values or their *H*_T_:*H*_O_ ratios) within the same taxonomic Order or Family. CR = critically endangered, EN = endangered, *H*_O_ = observed heterozygosity, *H*_T_ = reduced heterozygosity after *T* generations, LD = linkage‐disequilibrium, *N*_c_ = census population size, *N*_e_ = effective population size, *t* = generation time, *T* = the number of generations in 100 years, VU = vulnerable, *μ* = mutation rates.

Our proposed GD criterion for the Red List is illustrated in Figure 3 and, in principle, could be readily applied by any conservation organization that conducts status assessments given that the model parameters can be estimated from publicly available resources. For example, *H*_O_ could be estimated from population genomic datasets using a standardized workflow (our Nextflow pipeline is available at https://github.com/jyj5558/theta), and generation time is generally known from life history studies. *N*_e_ can either be estimated indirectly from census population size (*N*_c_) where *N*_e_ is crudely estimated from *N*_c_ (Frankham 1995; Palstra and Fraser 2012), or directly from population genomic data. For example, contemporary *N*_e_ can be estimated using the linkage‐disequilibrium‐based method (e.g., *currentNe*; Santiago et al. 2020) so long as practitioners recognize that genomes do not immediately register demographic changes (i.e., there is a lag time or drift debt; Patton et al. 2019; Pinto et al. 2024; Waples 1990).

Here, we provide initial recommendations for how GD could be used to help assign extinction risk categories (i.e., CR, EN, or VU). These novel recommendations will ultimately evolve, but we base them on an established idea: the rate of heterozygosity loss over time (Allendorf and Ryman 2002; Díez‐del‐Molino et al. 2018; Frankham, Bradshaw, and Brook 2014; Lande 1988; Lynch and Lande 1998). We suggest that conservation authorities rely primarily on the rate of GD loss to help determine the threat category, where the rate is defined as the ratio of *H*_T_ to *H*_O_. Secondarily, we propose that explicit *N*_e_ cutoffs be used to help ensure that populations with demographic characteristics that put them at high risk of genetic erosion are effectively evaluated. For example, species with long generation times (e.g., whales) will register GD changes too slowly with the *H*_T_:*H*_O_ ratio and thus are more appropriate to evaluate with *N*_e_.

If the generation time is long or if *N*_e_ cannot be reasonably estimated (e.g., because of uncertainty in *N*_c_), then we recommend using relative *H*_O_ thresholds to classify taxa into “Non‐threatened” or “Threatened” categories. Our rationale is that *H*_O_ varies among taxonomic categories (Figure 1) and that *H*_O_ is often associated with threatened status (Table S2, Figures S7 and S8), indicating that it often conveys valuable conservation signal. This relative *H*_O_ threshold would effectively provide conservation insurance by enabling assessments of elusive species where sufficient sample sizes for accurate *N*_e_ estimation are difficult or impossible to obtain (Waples 2024a). We propose that relative *H*_O_ cutoffs be used in a simple binary fashion (yielding assessments of threatened or not) because snapshot *H*_O_ values cannot determine whether low heterozygosity across the genome is due to recent genomic erosion (e.g., inbreeding) or to natural demographic events such as historic bottlenecks in species like the cheetah (O'Brien et al. 2017; Schmidt et al. 2023). Thus, the value of relative *H*_O_ should be discounted relative to the information contained in the *H*_T_:*H*_O_ ratio. We propose that species whose relative *H*_O_ is less than the maximum *H*_O_ of related species or populations (i.e., those in the same taxonomic Order or Family) that are already classified as CR, EN, or VU based on *their H*_T_:*H*_O_ ratios or effective population sizes. We propose a threshold associated with a maximum *H*_O_, rather than a mean or median value, because those measures of central tendencies will change each time a new *H*_O_ value is added (e.g., in a novel *H*_O_ assessment of a related species) whereas the maximum *H*_O_ will only increase and thus provides more stability to GD assessments.

In practice, it can be difficult to estimate credible *N*_e_ values in a standardized way across taxa. This is because *N*_e_ estimates depend on the approach employed (e.g., heterozygote excess, linkage disequilibrium, and temporal variance in allele frequencies), the number of variable loci, the number of samples, and other biological factors (e.g., reproductive skew) that vary across taxa (Waples 2024a). Thus, when possible we suggest the use of the *N*_e_/*N*_c_ ratio to estimate *N*_e_ (Waples 2024b) but recognize that *N*_e_ may need to be estimated with genomic approaches (e.g., linkage disequilibrium).

Our specific recommendations are shown in Figure 3. If the rate of heterozygosity loss is extreme (i.e., *H*_T_:*H*_O_ ≤ 0.95), we think this is sufficiently worrisome that it warrants a CR or EN categorization. In cases where the rate of heterozygosity loss is less extreme, perhaps due to the 100‐year time frame coupled with long generation times, we suggest supplementing the *H*_T_:*H*_O_ ratio with *N*_e_ as described with the Boolean operators in Figure 3. For instance, *H*_T_:*H*_O_ = 0.987 in the cheetah (*Acinonyx jubatus*) when using a 10% *N*_e_/*N*_c_ ratio (Table S6), which is relatively high. As a safeguard for species that have long generation times (6 years for the cheetah), the logic shown in Figure 3 and Table S6 suggests that if an additional evaluation revealed that *N*_e_ < 1000, then the cheetah should be assigned to the VU category. On the other hand, when using a 100% *N*_e_/*N*_c_ ratio, the cheetah's *H*_T_:*H*_O_ = 0.997 and *N*_e_ = 6517, thus categorizing it as LC. However, the cheetah's *H*_O_ is 0.00041, which is lower than the maximum (=0.00118 of *Panthera tigris jacksoni*) of genetically Threatened Canivora species (i.e., species of the Order Canivora which are assigned VU, EN, or CR according to the *H*_T_:*H*_O_ cutoff or *N*_e_ cutoff), and this would result in a Threatened determination for the cheetah.

**FIGURE 4:**
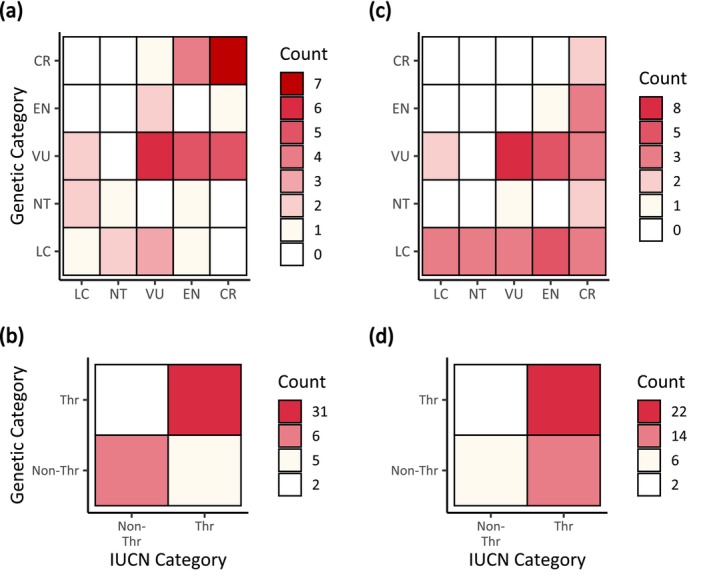
The comparison between original, official IUCN categories and hypothetical genetic categories based solely on the genetic criterion described in this study, whether full (a and c) or binary (b and d) plus “Data Deficient,” when the *N*_e_/*N*_c_ ratio was 10% (a and b) or 100% (c and d). Note that we assigned “VU” to Threatened species using the relative *H*_O_ threshold for purposes of illustration. CR = critically endangered, DD = data deficient, EN = endangered, LC = least concern, NT = near threatened, Thr = threatened, VU = vulnerable.

How do these general recommendations perform using real data? We tested these thresholds by using median census population size (*N*_c_) for Red List species when available or, alternatively, by employing *currentNe* to estimate *N*_e_ from the publicly available sequence data for “Data Deficient” species (i.e., those without Red List information) to illustrate how genomic data could be used when demographic or other data are lacking. Because the ratio of effective to census size varies over time (Ardren and Kapuscinski 2003; Wang et al. 2023) and across taxa (Palstra and Fraser 2012), we used two extreme ratios of *N*_e_/*N*_c_, 10% (Frankham 1995) and 100% (Wang et al. 2023) to illustrate the strictest and the most lenient assessments. We found that in many cases, the loss of heterozygosity over 100 years (“*H*_T_/*H*_O_” in Table S6) was <2.5% due to large census population sizes and/or long generation intervals, but two “Data Deficient” species were assigned “CR” due to small estimates of *N*_e_. When we used a 10% *N*_e_/*N*_c_ ratio, the overall trend of genetic categories was quite similar to that of the original IUCN categories (Figure 4a,b), but when a 100% *N*_e_/*N*_c_ ratio was used, genetic categorization was more lenient than the original categorization (Figure 4c,d). Thus, our GD recommendations can effectively supplement the original Red List categorization, especially given that the final (official) category is determined as the highest threat category determined across multiple assessment criteria (IUCN 2012). Further research will be needed to determine the most robust *N*_e_/*N*_c_ ratio or *N*_e_ estimator for any given lineage.

### 4.4 The Potential Role of GD in Green Status Conservation Assessments

IUCN's newer assessment scheme, the Green Score, is calculated as:

$$G=\frac{\sum_{S}W_{S}}{W_{F}\times N}\times 100$$

where *W*_S_ is the weight of a spatial unit (S; could be a geographic population, for example) defined as 0 (absent), 3 (present), 6 (viable state), or 9 (functional state); W_F_ is weight of the functional state (i.e., 9); and *N* is the number of spatial units (https://www.iucnredlist.org/assessment/measuring‐recovery‐green‐status‐species). The Green Score can address intraspecific, population‐level conservation status. This aspect of the Green Score is extremely valuable, but we think it could be enhanced with a “genetic diversity correction” because relative GD is associated with population productivity and adaptability. Explicitly, relative GD could be incorporated into the Green Score by weighting a population's GD against the average GD among related LC species (to correct for inherent biases in GD across organismal groups, as shown in Figures S2 and S3 and in Garner, Rachlow, and Hicks 2005). Incorporating this suggestion into the existing Green Score equation would give the equation:

$$G=\frac{\sum_{S}\left(W_{S}\times \frac{{\mathrm{GD}}_{S}}{{\mathrm{GD}}_{\mathrm{LC}}}\right)}{W_{F}\times N}\times 100$$

where GD_S_ is the GD level of a spatial unit and GD_LC_ is the average GD level of “LC” species or populations (the maximum value of GD_S_/GD_LC_ would thus be capped at 1).

There are several reasonable GD parameters that would be suitable for use in our suggested modifications of the Green Score calculator. For example, *H*_O_ estimates are alluring for the same reasons we recommend its use for estimating extinction risk: *H*_O_ is related to existing IUCN categories and *H* has a solid theoretical foundation. However, if *N*_e_ were used as the GD metric in the formula, this approach could accommodate two CBD indicators that Hoban et al. (2020) suggested for the CBD's post‐2020 Global Biodiversity Framework—“the number of populations [or breeds] within species with an effective population size (*N*_e_) above 500 compared to the number below 500” and “the number of species and populations in which genetic diversity is being monitored using DNA based methods.” We could further pursue Goal A in CBD's Post‐2020 Global Biodiversity Framework (i.e., “maintaining at least 90% of GD of all species”) by targeting the ratio of (∑GD_S_)/(GD_LC_ × *N*) ≥0.9 for each species. Regardless of the selected metric, our intent is to illustrate that the Green Score could be further enhanced by explicitly addressing GD, and that these enhancements would help conservationists better estimate prominent biodiversity indicators used by international organizations like the CBD (Box 1).

BOX 1 **Potential incorporation of a novel GD criterion into the Green Score**

Here, we provide an example application of the modified Green Score for California towhee (*Melozone crissalis*) and Inyo California towhee (*M. c. eremophilus*). We used their *H* values (Black et al. 2023) and compared those to *H* values of Least‐Concern birds (Brüniche‐Olsen et al. 2021). California towhee was sampled from nine geographic sites where *H* values were [0.00169, 0.00187, 0.00194, 0.00206, 0.00206, 0.00208, 0.00218, 0.00246, 0.00249] and the Inyo California towhee was sampled from a single population whose *H* was 0.00180, compared to an average *H* value of 10 Least‐Concern Passerine species of 0.00355 (Brüniche‐Olsen et al. 2021). To focus on the influence of GD correction, we assumed that all the available populations (spatial units in the Green Score formula) were sampled for each taxon and that all the California towhee populations are in a functional state whereas the Inyo California towhee population is in a viable state. Without the GD correction, the Green Score of California towhee would be:

$$\frac{12\times \left(1+1+1+1+1+1+1+1+1\right)}{12\times 9}\times 100$$

=100.

whereas the GD‐corrected Green Score of California towhee would be

12×0.00169+0.00187+0.00194+0.00206+0.00206+0.00208+0.00218+0.00246+0.002490.0035512×9×100

=58.936.

Without the GD correction, the Green Score of the Inyo California towhee would be

$$\frac{9\times 1}{12\times 1}\times 100$$

=75

whereas the GD‐corrected Green Score of Inyo California towhee would be

9×0.001800.0035512×1×100

=38.028

Thus, the GD correction reduced the Green Scores by ~40%–50%, and provides a richer and more nuanced insight into the status of these two taxa.

### 4.5 Limitations

GD worked well for conservation assessments in this study, but we acknowledge some potential limitations. First of all, genomic resequencing data is not always available but we note this is a potential criticism of *any* assessment criteria, including those already in use by IUCN and other organizations. Secondly, some species seem to thrive even with low GD (e.g., Femerling et al. 2023). However, it is also true that some species seem to thrive even when they score poorly with existing assessment criteria, such as small population sizes or small geographic ranges (e.g., bottlenecked invasive species). Thirdly, there is the potential of unrecognized “drift debt,” the time‐lag impact of genetic drift akin to the “extinction debt,” that has not yet manifested itself in species with long generation intervals (Pinto et al. 2024). Finally, our suggestions are overly simplistic. Additional genomic assessments (e.g., genetic load, genomic offset, and pangenomics) could ultimately be incorporated to produce a more comprehensive GD criterion of the future, but then the challenge to explain the criterion to non‐geneticists becomes even more daunting.

### 4.6 Conclusion

Our analyses show that the five conservation criteria currently used by the IUCN Red List (census population size, demographic trajectory, geographic range size, a combined index of population size and geographic range size, and associated quantitative analyses) indirectly capture GD. However, many species on IUCN's Red List are “Data Deficient” because parameters like census population size or demographic trajectory are extremely difficult to estimate. We think that GD could become valuable as an additional criterion for conservation assessments, in large part because GD can be more easily and inexpensively evaluated than census size or demographic trajectory and can be estimated directly from public sequence databases that are expanding rapidly. We reiterate that neither *H* nor any other GD criterion should replace any existing assessment criteria. Instead, we think a GD criterion should be another point of emphasis (e.g., as a baseline level or how it trends over time) in a holistic evaluation of conservation threats (Red List) or successes (Green Status). We have illustrated our ideas using mammalian data, but these ideas are applicable to other branches on the tree of life.

We hope our proof‐of‐concept analyses and suggestions for a novel GD criterion will provide a springboard for further discussion. Regardless of whether the scientific community embraces our specific recommendations, we think conservationists would do well to explicitly assess GD metrics as part of a comprehensive evaluation of each species and there are a number of conservation genetics organizations (e.g., the IUCN Conservation Genetics Specialist Group) poised to help. Our study outlines the theoretical and empirical justification for a new GD criterion, a bioinformatic pipeline for estimating GD from publicly‐available population genomic data, an analytical framework, and explicit recommendations for use by conservation authorities. Contemporary GD is critical to population persistence, and we hope that institutional authorities are prescient enough to recognize that exponentially expanding sequence repositories (Karasikov et al. 2024; Offord 2024) are a rich source of biological insight; we only need to take advantage of them.

## Conflicts of Interest

We declare no competing interests, but note that JAD is a member of IUCN's Species Survival Commission (SSC) North America and that JAD and ABO are members of the IUCN SSC Conservation Genetics Specialist Group.

## Supporting information

SupInfo

**Dataset S1.** Information on the species analyzed in this study. The species information (*N* = 82) includes taxonomic information (species names according to different data sources, taxonomic Order, and taxonomic Family), the Red List information (category, population trend, and geographic range), eco‐evolutionary factors (trophic level, habitat type, habitat breadth, and body mass; note that habitat type is not used in the statistical analyses due to its biased distribution), estimated values of GD metrics (nucleotide diversity, Watterson’s Theta, Tajima’s *D*, heterozygosity per population, heterozygosity per individual, *F*100kb = *F*_ROH>100kb_, *F*1Mb = *F*_ROH>1Mb_), and genomic statistics (Reference genome—assembly level, contig N50, scaffold N50; resequencing data—sample size, sequencing chemistry, mean sequencing depth per individual, average of depth per species, and standard deviation of depth per species).

**Dataset S2.** The “IUCN” dataset used for the PGLS between IUCN categories and GD metrics. This dataset includes all the species having their own Red List assessment, excluding “Data‐Deficient” species (*N* = 72); Table S1 is a subset of Dataset S1.

**Dataset S3.** The “EcoEvo” dataset used for the PGLS between eco‐evolutionary factors and GD metrics. This dataset includes all the species having their own eco‐evolutionary factors in the COMBINE database (*N* = 63); Table S2 is a subset of Dataset S1.

**Table S6.** Results of applying the proposed genetic criterion with the effective population sizes based on the median population size from the Red List or estimated using “*currentNe*.” Information on mutation rates and generation times were obtained from previous literature and the COMBINE database. The original IUCN categories were shown side‐by‐side with the genetic categories for comparison. The column “genetic_category_beforeHcutoff_10%Nc” represents genetic categories after the *H*_T_:*H*_O_ cutoff and *N*_e_ cutoff based on 10% *N*_e_/*N*_c_ ratio but before applying the relative *H*_O_ value cutoff. The column “genetic_category_beforeHcutoff_100%Nc” represents genetic categories after applying the *H*_T_:*H*_O_ cutoff and *N*_e_ cutoff based on 100% *N*_e_/*N*_c_ ratio but before applying the relative *H*_O_ value cutoff. The column “genetic_category_final_10%Nc” represents genetic categories after applying the *H*_T_:*H*_O_ cutoff and *N*_e_ cutoff based on 10% *N*_e_/*N*_c_ ratio and the relative *H*_O_ value cutoff. The column “genetic_category_final_100%Nc” represents genetic categories after applying the *H*_T_:*H*_O_ cutoff and *N*_e_ cutoff based on 100% *N*_e_/*N*_c_ ratio and the relative *H*_O_ value cutoff. When a species category was changed due to the relative *H*_O_ value cutoff, it was indicated with yellow color (for the genetic criterion based on 10% *N*_e_/*N*_c_ ratio) or orange color (for the genetic criterion based on 100% *N*_e_/*N*_c_ ratio). The species’ heterozygosity values whose genetic category was changed after applying the relative *H*_O_ value cutoff are italicized. The maximum heterozygosity values of genetically Threatened species (i.e., species of VU, EN, or CR before applying the relative *H*_O_ value cutoff) within each taxonomic order are bolded. The genetically Threatened species with the maximum heterozygosity value could be different between 10% *N*_e_/*N*_c_ ratio and 100% *N*_e_/*N*_c_ ratio used, so we filled the cell with yellow (for the case of 10% *N*_e_/*N*_c_ ratio) or orange (for the case of 100% *N*_e_/*N*_c_ ratio).

**Table S7.** NCBI accession numbers for reference genomes and NCBI BioProject numbers for resequencing data are listed along with information on the specific population of each species (geographic locality).

## Data Availability

Genomic data collected from the NCBI (species, population, accession numbers for reference assembly, BioProject numbers for WGR, etc.) are reported in Table S7. Data for statistical analyses were shared as Datasets S1–, S3. Bash, Python, R, and Nextflow scripts used in the study were uploaded to the GitHub repository: https://github.com/jyj5558/theta.
