# Supplementary material for: Genomic Diversity as a Key Conservation Criterion: Proof‐of‐Concept From Mammalian Whole‐Genome Resequencing Data
Source: Evol Appl. 2024 Sep 10;17(9):e70000. doi: 10.1111/eva.70000 (PMC11386325; doi:10.1111/eva.70000)
Supplement: Supplementary file 1 — SupInfo [file EVA-17-e70000-s002.docx]

**Supplementary Information for**

Genomic diversity as a key conservation criterion: proof-of-concept from mammalian whole-genome resequencing data

Jong Yoon Jeon^1^, Andrew N. Black^1,2^, Erangi J. Heenkenda^1^, Andrew J. Mularo^3^, Gina F. Lamka^4^, Safia Janjua^1^, Anna Brüniche-Olsen^5^, John W. Bickham^6^, Janna R. Willoughby^4^, and J. Andrew DeWoody^1,2^

^1^ Department of Forestry and Natural Resources, Purdue University, West Lafayette, IN 47907, USA

^2^ Western Association of Fish and Wildlife Agencies, Boise, Idaho, USA

^3^ Department of Biological Sciences, Purdue University, West Lafayette, IN 47905, USA

^4^ College of Forestry, Wildlife, and Environment, Auburn University, Auburn, AL 36849, USA

^5^ Department of Biology, University of Copenhagen, Ole Maaløes Vej 5, 2200 Copenhagen N, Denmark

^6^ Department of Ecology and Conservation Biology, Texas A&M University, College Station, TX 77840, USA

* J. Andrew DeWoody

Email: [dewoody@purdue.edu](mailto:jeon96@purdue.edu)

Supplementary Information Text

**Appendix A1. Overall workflow of this study**


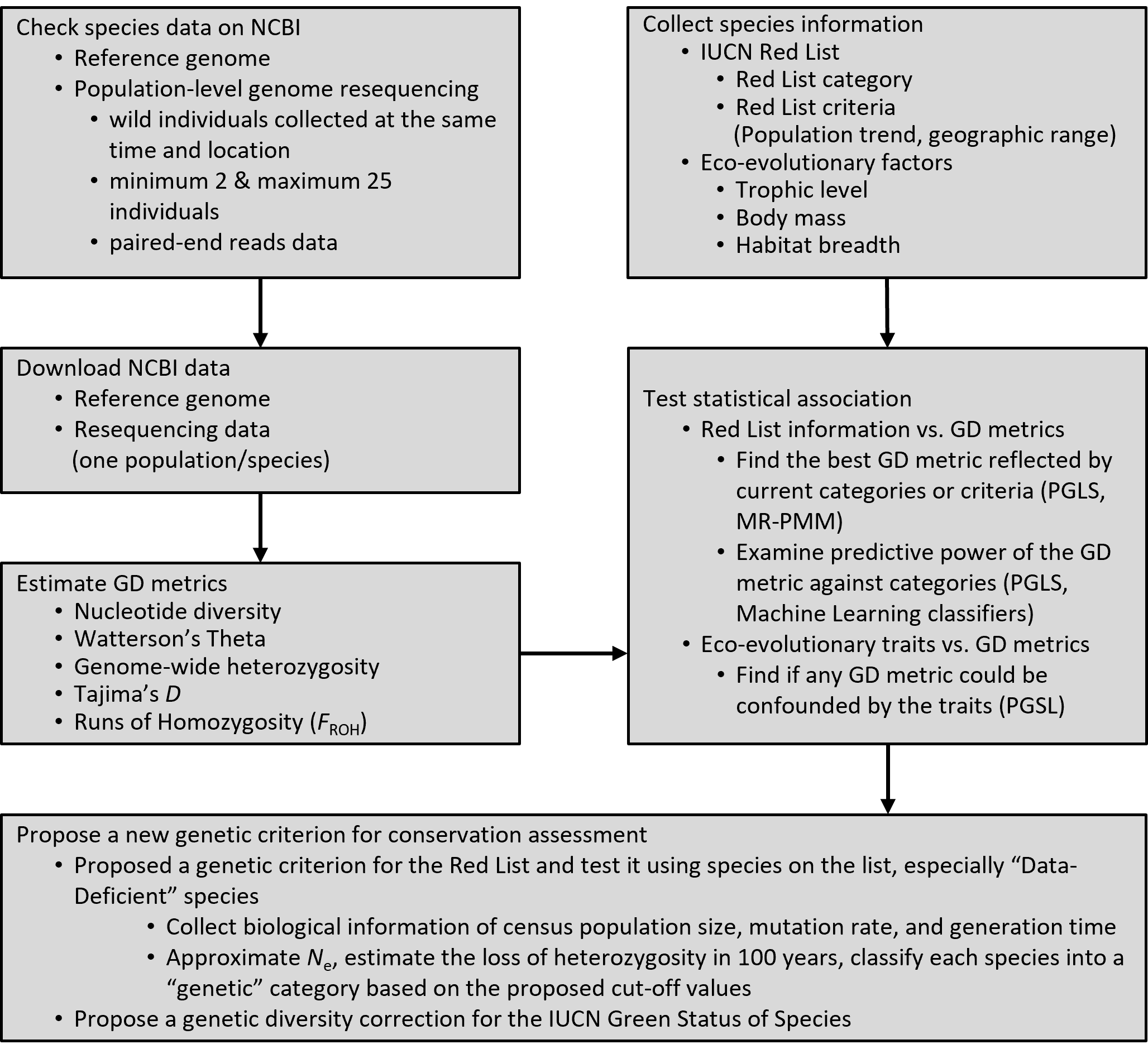


Appendix Figure Fig. A1. Overall workflow of the study from data collection (steps 1–3) to statistical analyses (step 5) and the genetic criterion proposal (step 6). See Methods for details of each step. Abbreviations: NCBI - National Center for Biotechnology Information, GD - Genetic/Genomic diversity, PGLS - Phylogenetic Generalized Least Squares, MR-PMM - Multi-Response Phylogenetic Mixed Modeling.

**Appendix A2. Technical artifact control**

Technical artifacts (i.e., different assembly level, sequencing chemistry, sample sizes, contig N50, scaffold N50, the average of read depths, and standard deviation of read depths) could potentially skew GD metrics, so we included them for the quality control in the main statistical analyses. All potential technical factors were subjected to a Multi Factor Analysis using R package ‘FactoMineR’^1^ as there was no correlation among the 7 technical factors. Similar factors were grouped and their contributions to resultant dimensions are summarized in Appendix Table A1. We also tested for correlation between individual read depth and individual heterozygosity using Pearson’s test but found no significant correlation.

We included the first four dimensions (explained ~80% of variance; Dimension1 - 27.70%, Dimension 2 - 21.14%, Dimension 3 - 18.84%, Dimension 4 - 11.76%) as control factors in the main statistical analyses. The Dimension 1 and Dimension 2 represented contiguity of reference genomes and sample size of resequencing data, respectively. We did not include each variable independently because too many variables compared to the sample size reduces statistical power^2^.

**Appendix Table A1.** Proportional contributions of each technical factor to the four dimensions considered in our Multi Factor Analysis. Avg. = Average, Stdev. = Standard deviation. Each Dimension column sums to 100%.

| Technical factor group | Dimension 1 | Dimension 2 | Dimension 3 | Dimension 4 |
| --- | --- | --- | --- | --- |
| Assembly level | 42.60 | 8.81 | 53.44 | 8.03 |
| Contig N50, Scaffold N50 | 42.17 | 2.62 | 1.51 | 8.21 |
| Sequencing chemistry | 7.43 | 2.37 | 38.91 | 0.06 |
| Sample size | 0.87 | 56.68 | 1.06 | 11.73 |
| Avg. Depth of WGR reads,  Stdev. Depth of WGR reads | 6.93 | 29.52 | 5.08 | 71.98 |

Appendix A3. Statistical tests for controlling phylogenetic influence between eco-evolutionary factors and genomic diversity (GD) metrics

We tested for associations between eco-evolutionary factors and genomic diversity (GD) metrics to account for biological traits that might confound the focal relationship between Red List status and GD. We predicted that species at higher trophic levels would have lower GD values because they usually maintain smaller population sizes and are more frequently disturbed by human influence^3–5^.

Three factors representing different eco-evolutionary attributes (trophic level, body mass, and habitat breadth) were collected from the COMBINE database^6^, where we relied on data from sister taxa if focal taxa data were unavailable. Statistical significance was tested by Phylogenetic Generalized Least Squares (PGLS) with GD as the dependent variable.

Models with trophic level as an independent variable could not significantly explain Watterson’s Theta (*θ*_W_), Tajima’s *D* (*D*), or *F*_ROH>1Mb_ (*F*1Mb). The trophic level model did significantly explain *F*_ROH>100kb_ (*F*100kb) (*F*_6,56_ = 2.433, *p* = 0.037, adjusted *R*-squared = 0.122, 𝜆 = 0 with 95% CI = NA–0.676), but not when technical factors were ignored (*F*_2,56_ = 2.094, *p* = 0.133, partial *ω*^2^ = 0.034; Appendix Figure A1 and Supplementary Table S2). Dimension 1 was the significant variable of this model (*F*_1,56_ = 9.178, *p* = 0.004, partial *ω*^2^ = 0.115). Body mass models were not significantly associated with the independent variables *θ*_W_, *D*, *F*100kb, or *F*1Mb. Results of all the habitat breadth models were also non-significant. Therefore, there were no further confounding factors to be controlled for in the IUCN conservation status-GD relationship, other than the phylogenetic signal we accounted for in the PGLS modeling.

GD was not significantly explained by trophic level, body mass, or habitat breadth. This was somewhat surprising, as relationships with GD have been reported in the literature (e.g., ^5,7,8^). That being said, our use of PGLS approaches should account for the influence of phylogenetic relatedness in regression modeling. For example, even if GD values of carnivores and herbivores are different between two trophic groups, PGLS will reduce the amount of information explaining the difference by treating information from closely related species as correlated by moderating the slope of the regression line. Thus, phylogenetically explainable variables should already be controlled for in PGLS, in contrast to the Red List categories which are distributed among species.

The distribution of data associated with reduced dimensions can help identify influential variables and outlier characteristics. We plotted the distribution of all the estimated GD metrics, conservation criteria and eco-evolutionary drivers in a Multi Factor Analysis (MFA) using R package ‘FactoMineR’ with the first two dimensions (Appendix Figure A2). Continuous variables, including GD metrics, were standardized in the MFA. Data points were colored by *θ*_W_ value and shaped by IUCN full category. Dimension 1, composed primarily of GD metrics (but not eco-evolutionary factors), indicates that species with higher mean *θ*_W_ are mostly those categorized as “LC” by IUCN.

**
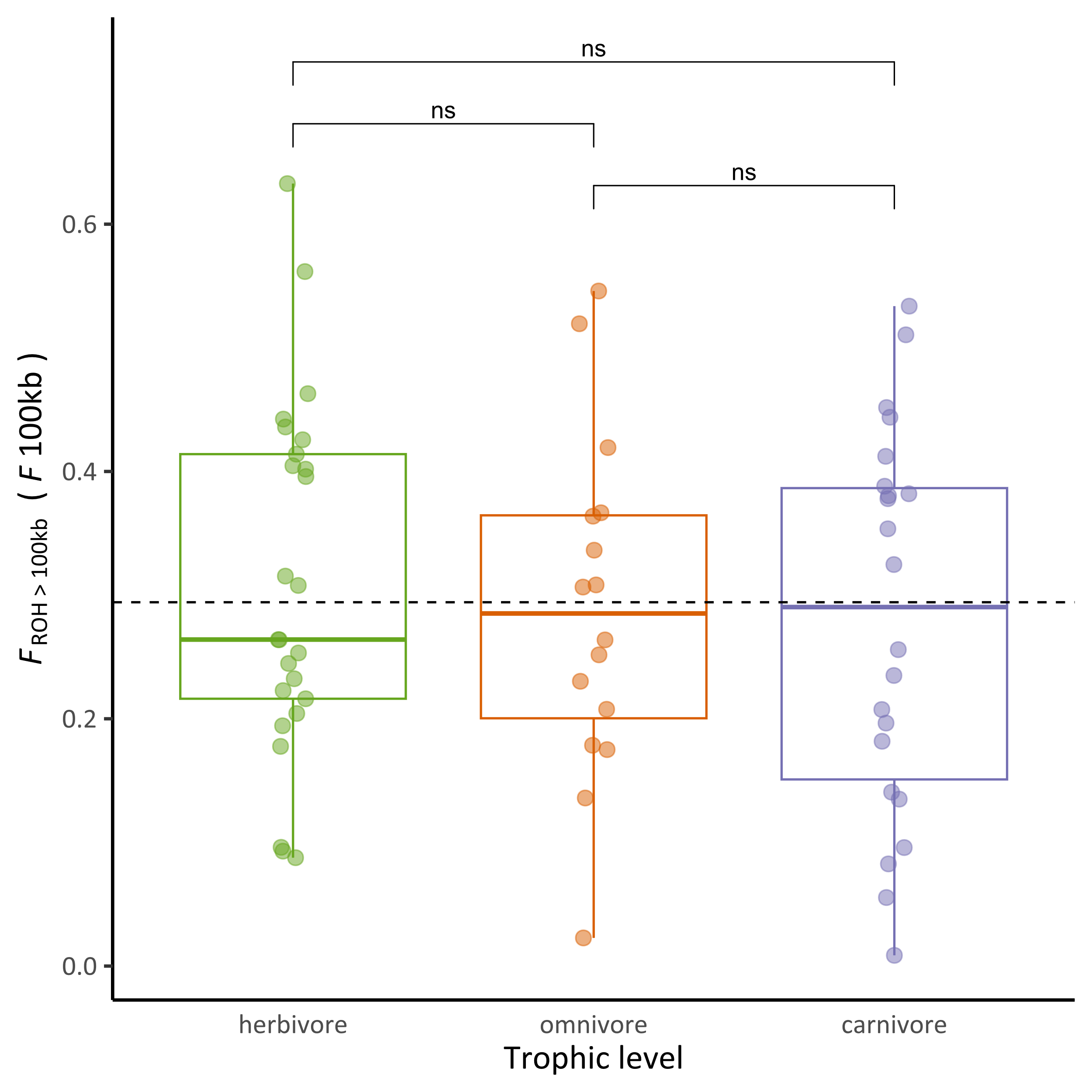
**

**Appendix Figure A2.** A box plot of arcsine-transformed *F*_ROH > 100kb_ against trophic level. Trophic level categories were compared to each other, but no differences between categories were significant. The boxes represent the range between the first and the third quartile (interquartile range, IQR) with the median line inside. The whiskers above and below the box represent the largest and smallest values within 1.5 IQR, respectively. Dashed line indicates the overall mean value.


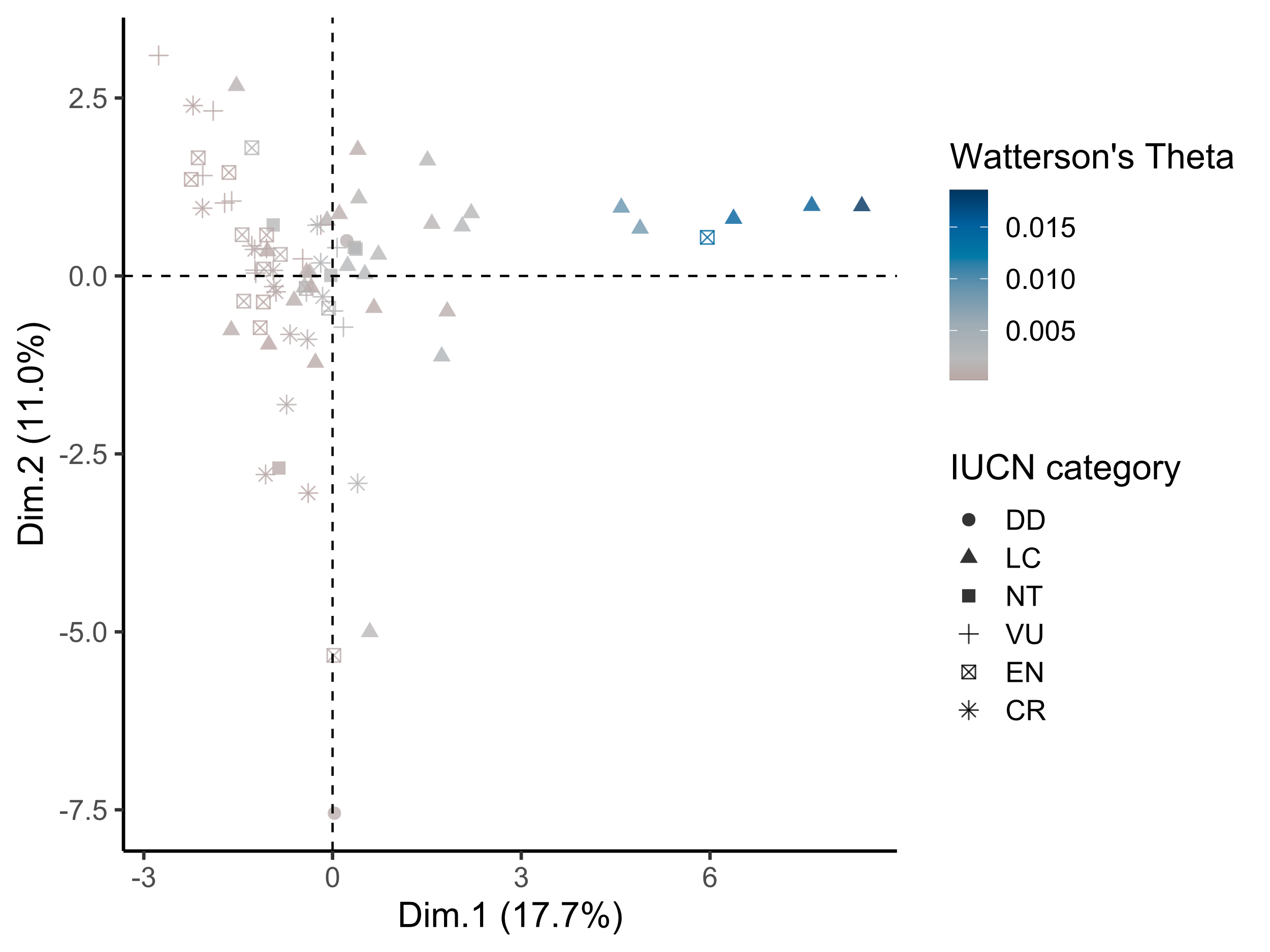


**Appendix Figure A3.** A Multi Factor Analysis plot of all 82 species analyzed by standardizing all variables of interest, including GD estimates, IUCN Red List assessment criteria, and eco-evolutionary drivers. Species were colored by Watterson’s Theta values (with a mid-point at the overall mean heterozygosity) and shaped by IUCN categories.

Appendix A4. Statistical test results between individual Red List assessment criteria and genomic diversity (GD) metrics

Two individual Red List assessment criteria, “population trend” and “geographic range”, were examined their association with GD metrics in place of the IUCN categorization. We ran Phylogenetic Generalized Least Squares models as described in the main text. Detailed models and results are listed in Supplementary Table S2.

Among models of “population trend” in place of IUCN categorization, the model with *F*100kb were significant, but not *F*100kb as an individual factor (Appendix Figure A4). Dim.1, consisting primarily of Assembly level and contig/scaffold N50s, was the significant variable in this case. Models with *D* and *F*1Mb were non-significant with also non-significant independent variables of interest. Using “geographic range” as an independent variable of interest, only *F*1Mb showed significance as an individual factor (Appendix Figure A5). Note that we could not estimate *F*1Mb in some species because of low contiguity of their reference genome and so could not determine if the absence of detection of long ROHs was due to their true (biological) absence or if absence of detection was due to technical factors such as being scattered across contigs.


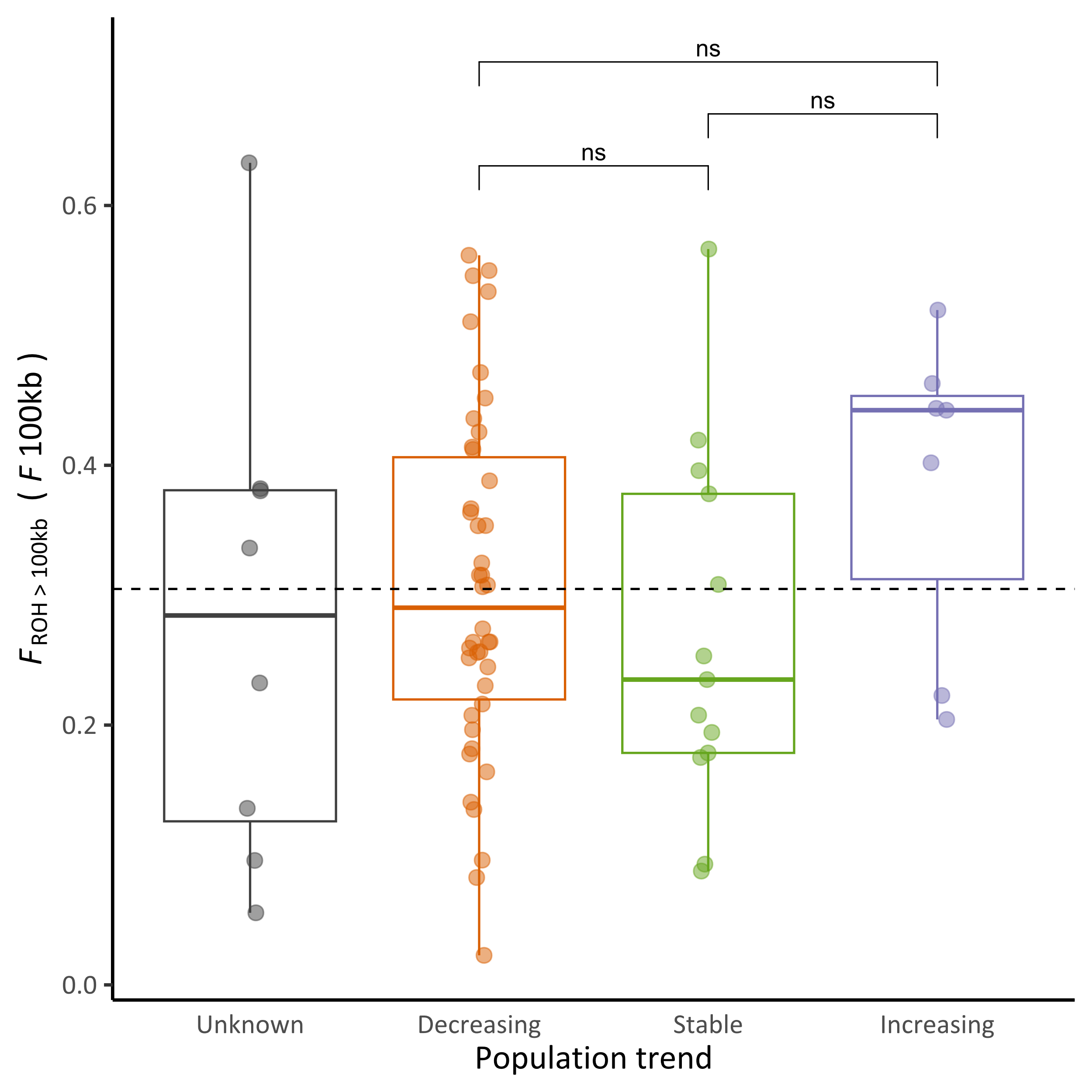


**Appendix Figure A4.** A box plot of arcsine-transformed *F*_ROH > 100kb_ against population trend. Statistical difference among population trend categories was assessed using a Kruskal-Wallis test (*p* = 0.18). Population trend categories were compared to each other and the significance is shown (ns: non-significant). The boxes represent the range between the first and the third quartile (interquartile range, IQR) with the median line inside. The whiskers above and below the box represent the largest and smallest values within 1.5 IQR, respectively. Dashed line indicates the overall mean value.

**
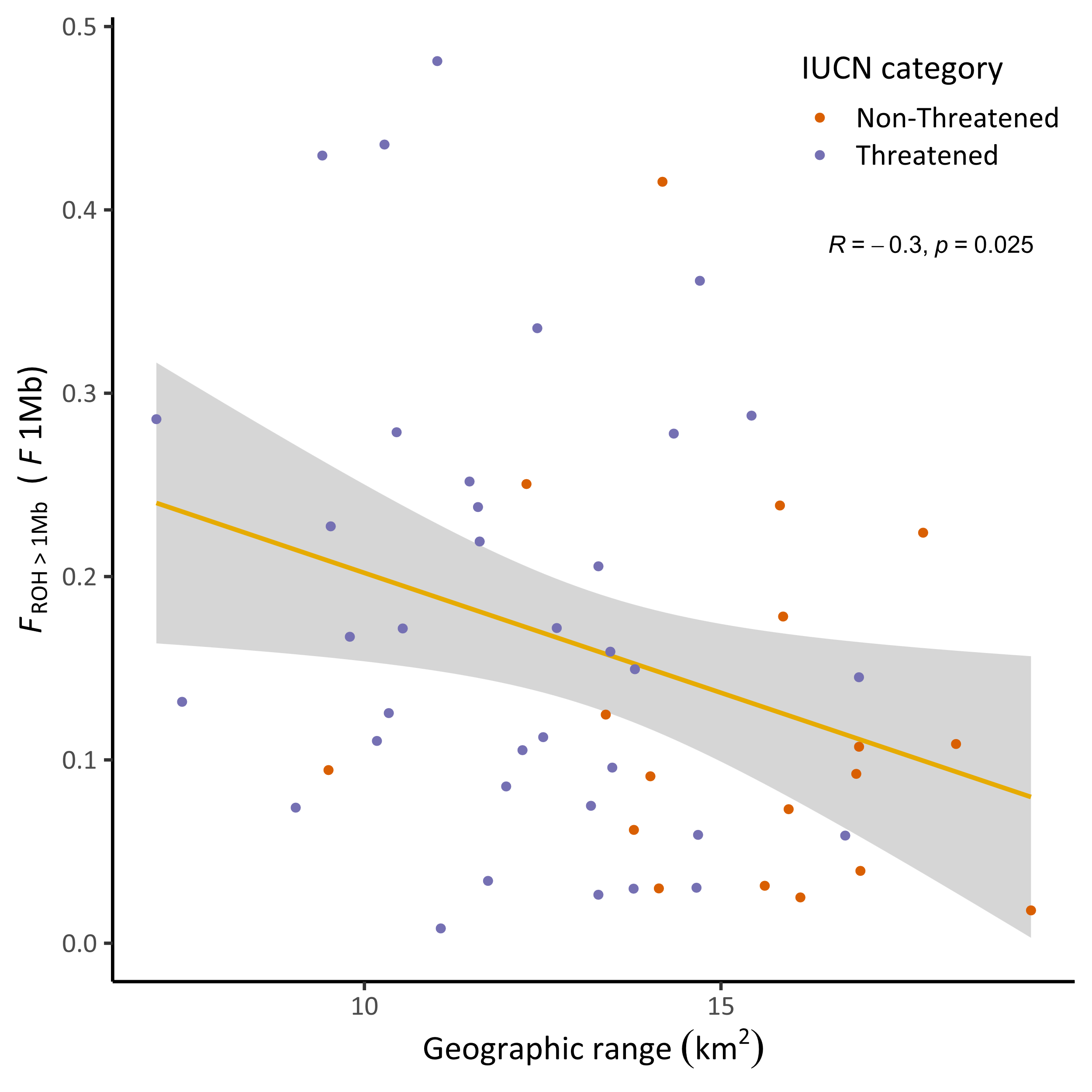
 Appendix Figure A5.** A scatter plot of arcsine-transformed *F*_ROH > 1Mb_ against geographic range. Geographic range is significantly negatively correlated with *F*_ROH > 1Mb_. A linear regression line is fitted, and its correlation coefficient and significance level are shown. The standard error interval of the line is shaded in grey.

**Supplementary Information Figures and Tables**


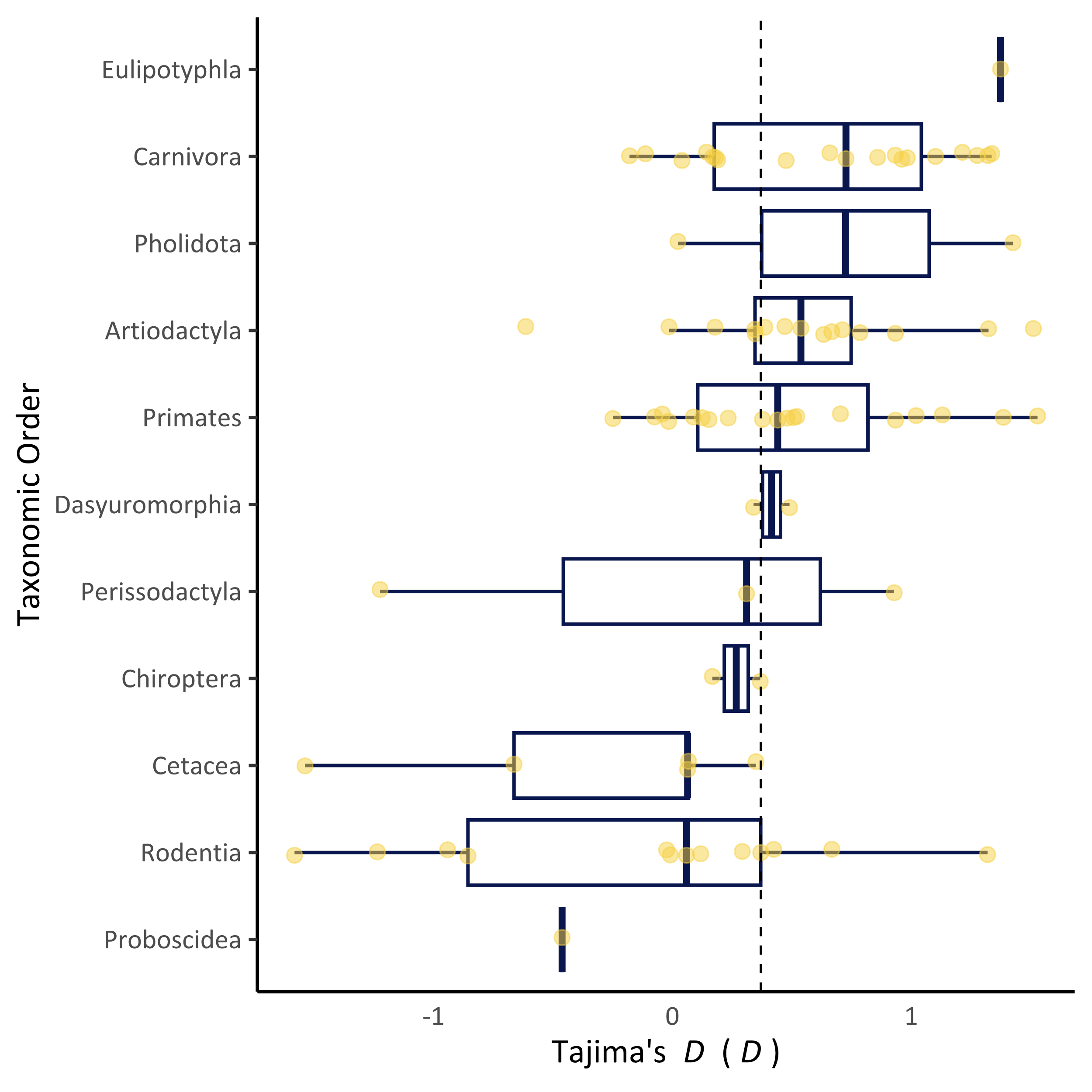


Fig. S1. A box plot of Tajima’s *D* by taxonomic Order. Taxonomic Orders are arranged by descending median value of Tajima’s *D*. The boxes represent the range between the first and the third quartile (interquartile range, IQR) with the median line inside. The whiskers above and below the box represent the largest and smallest values within 1.5 IQR, respectively. Dashed line indicates the overall mean value.

**
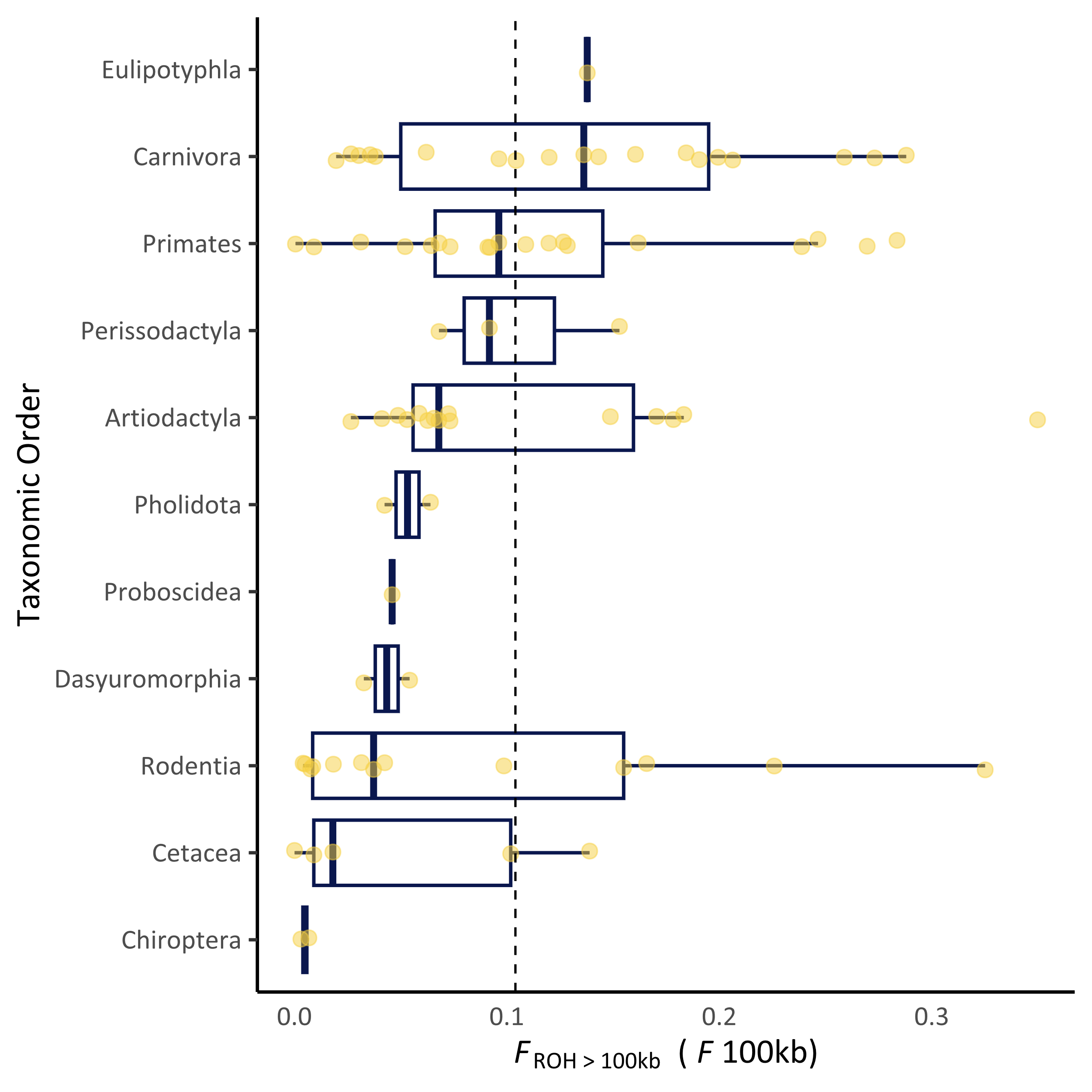
Fig. S2.** A box plot of *F*_ROH > 100kb_ by taxonomic Order. Taxonomic Orders are arranged by descending median value of *F*_ROH > 100kb_. The boxes represent the range between the first and the third quartile (interquartile range, IQR) with the median line inside. The whiskers above and below the box represent the largest and smallest values within 1.5 IQR, respectively. Dashed line indicates the overall mean value.

**
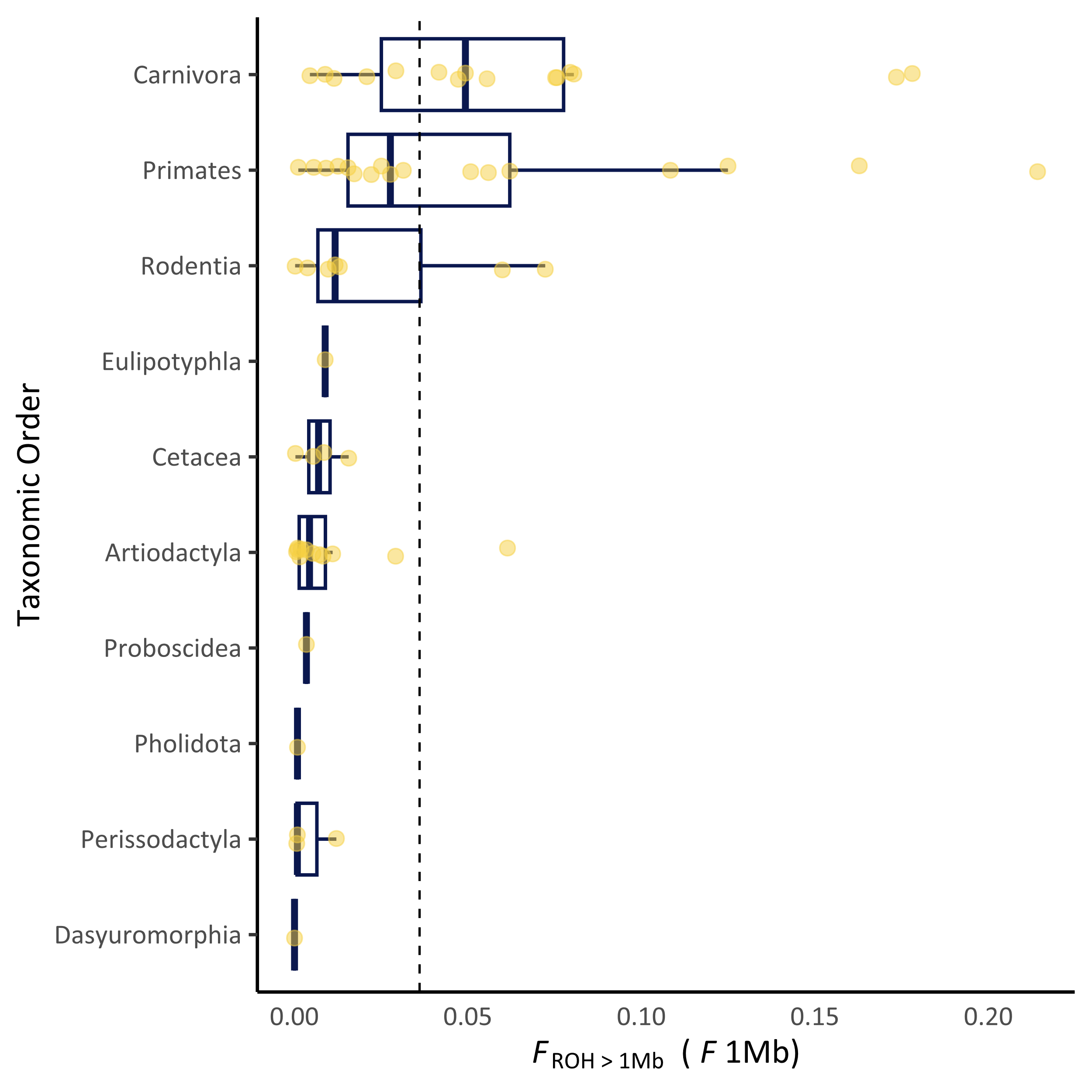
Fig. S3.** A box plot of *F*_ROH > 1Mb_ by taxonomic Order. Taxonomic Orders are arranged by descending median value of *F*_ROH > 1Mb_. The boxes represent the range between the first and the third quartile (interquartile range, IQR) with the median line inside. The whiskers above and below the box represent the largest and smallest values within 1.5 IQR, respectively. Dashed line indicates the overall mean value.


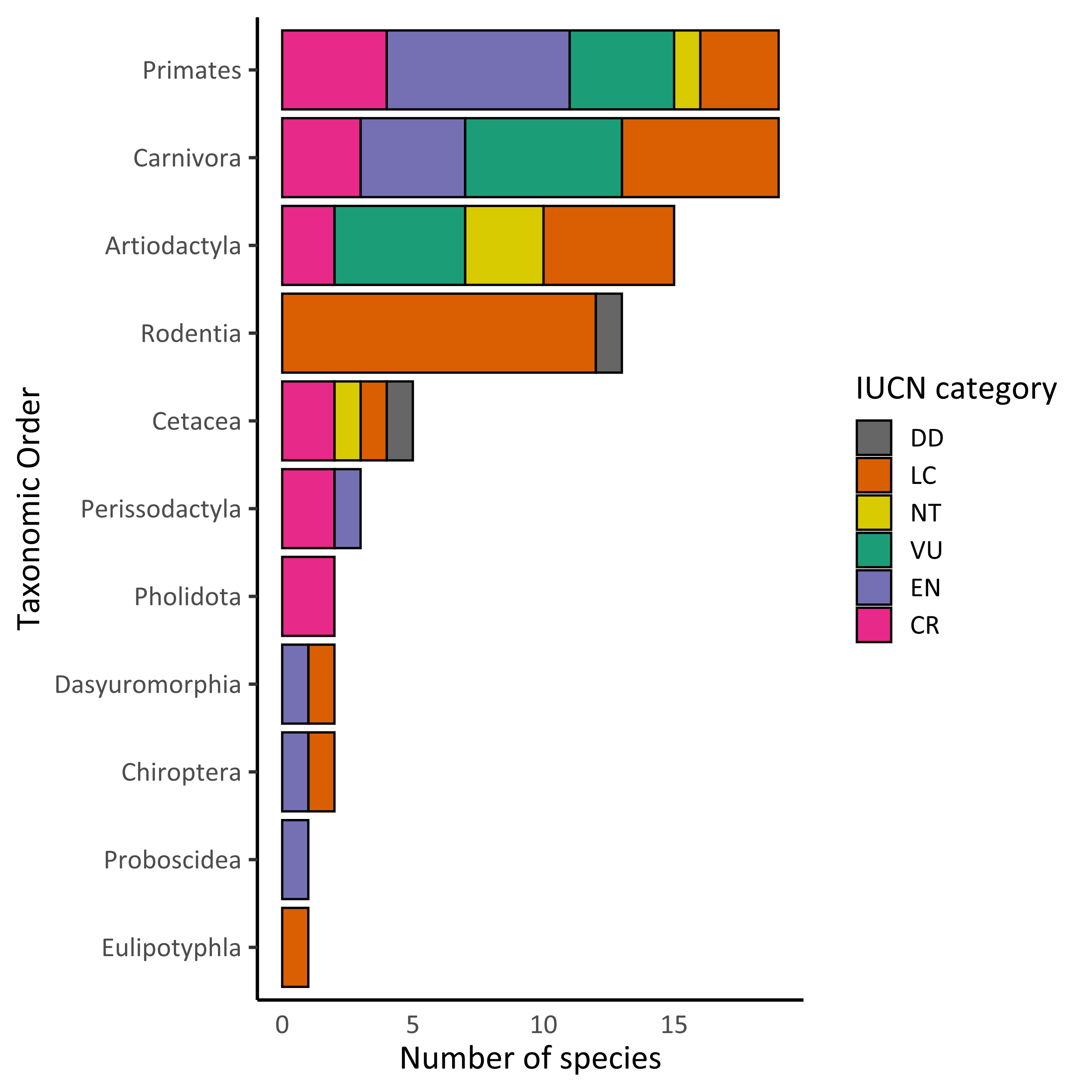
**Fig. S4.** A stacked bar plot of IUCN full category counts by taxonomic Order. IUCN full categories are color-coded. Taxonomic Orders are arranged by descending number of species.


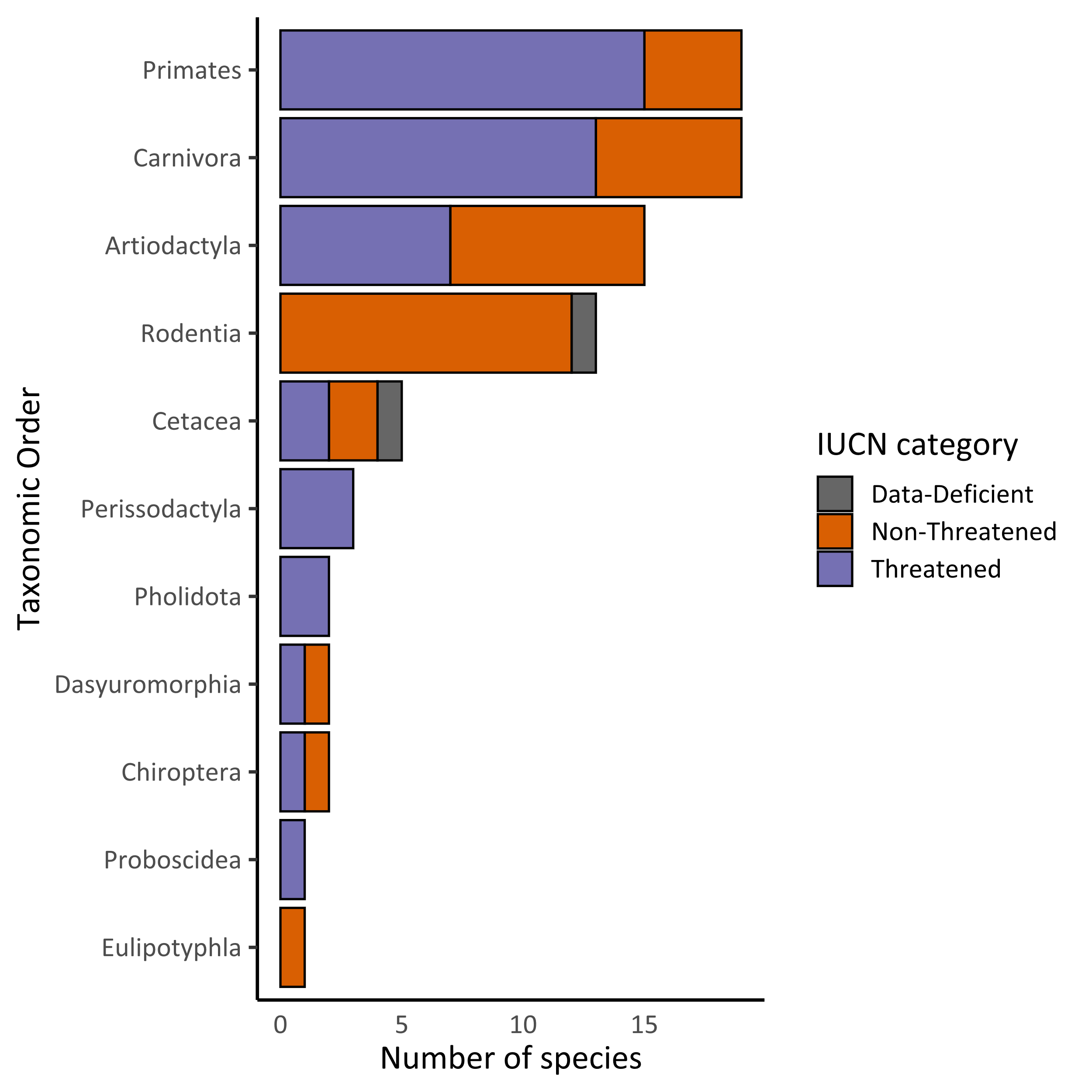
Fig. S5. A stacked bar plot of IUCN Threatened/Non-Threatened category counts, plus “Data-Deficient”, by taxonomic Order. IUCN Threatened/Non-Threatened categories, plus “Data-Deficient”, are color-coded. Taxonomic Orders are arranged by descending number of species.


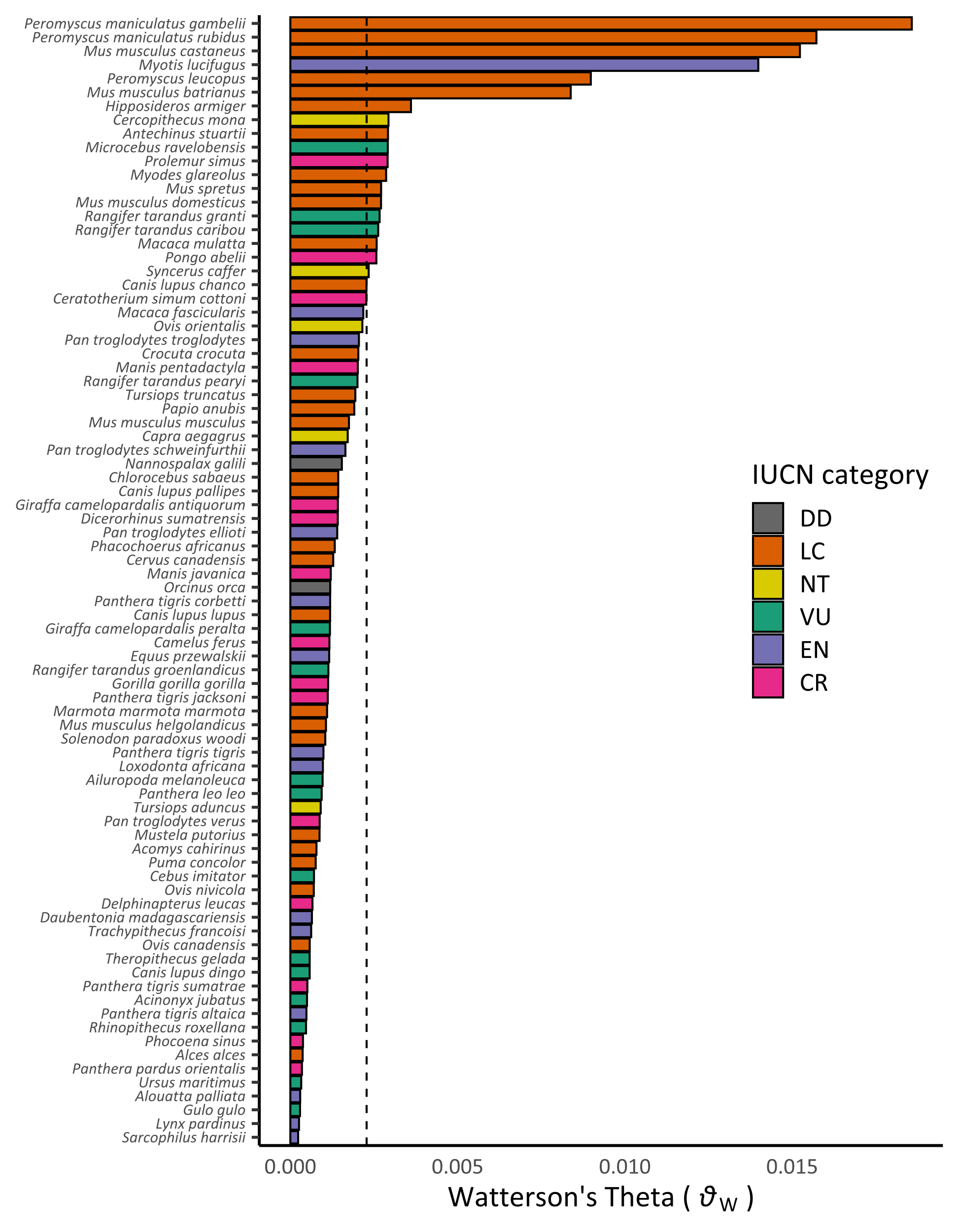
Fig. S6. A bar plot of Watterson’s Theta by species. Species are arranged by descending value of Watterson’s Theta and colored by IUCN full categories. Dashed line indicates the overall mean value. Species names (according to NCBI) are shown on the y-axis.


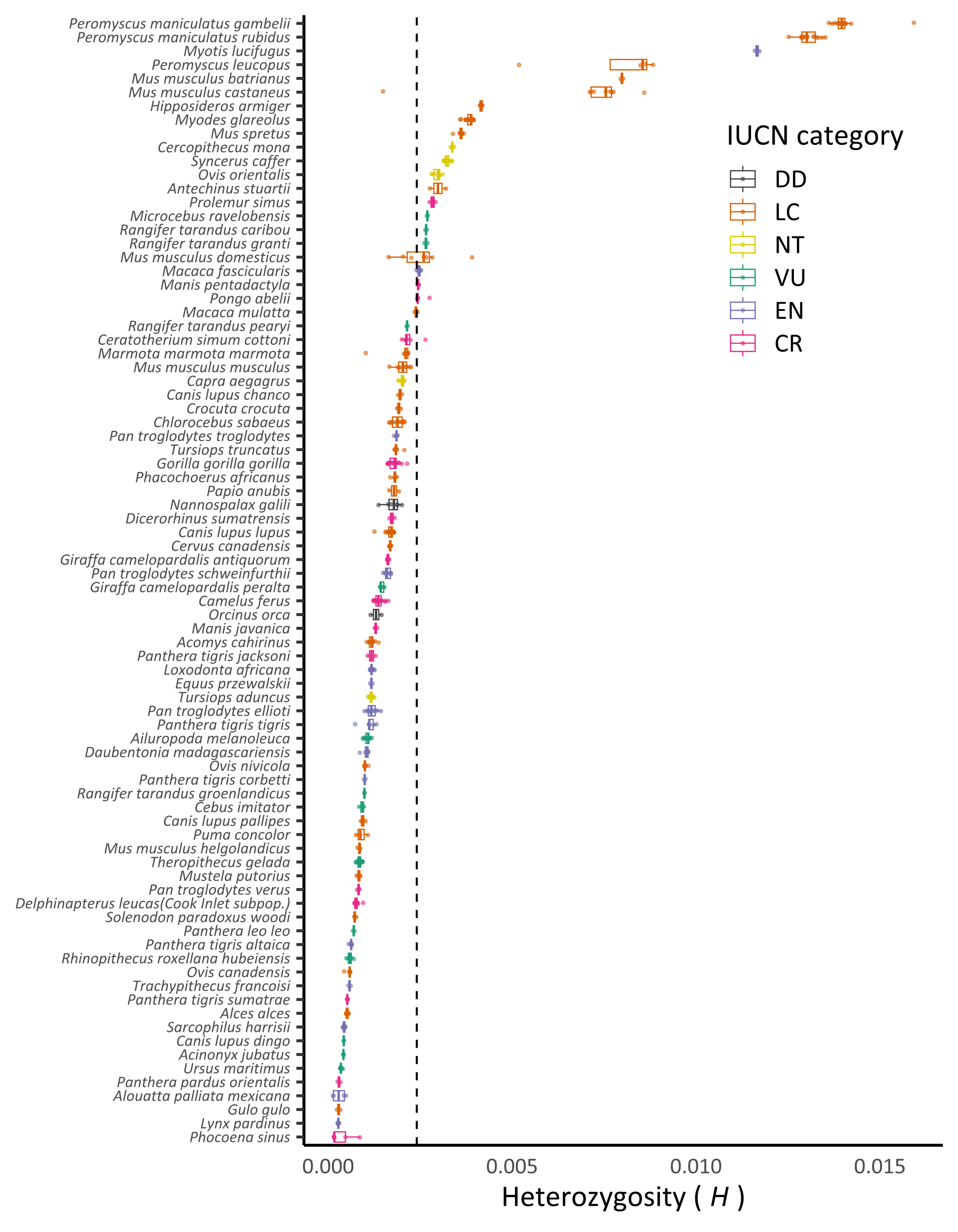
Fig. S7. A box plot of heterozygosity by species. Species are arranged by descending median value of heterozygosity and colored by IUCN full categories. The boxes represent the range between the first and the third quartile (interquartile range, IQR) with the median line inside. The whiskers above and below the box represent the largest and smallest values within 1.5 IQR, respectively. Dashed line indicates the overall mean value. Species names (according to NCBI) are shown on the y-axis.


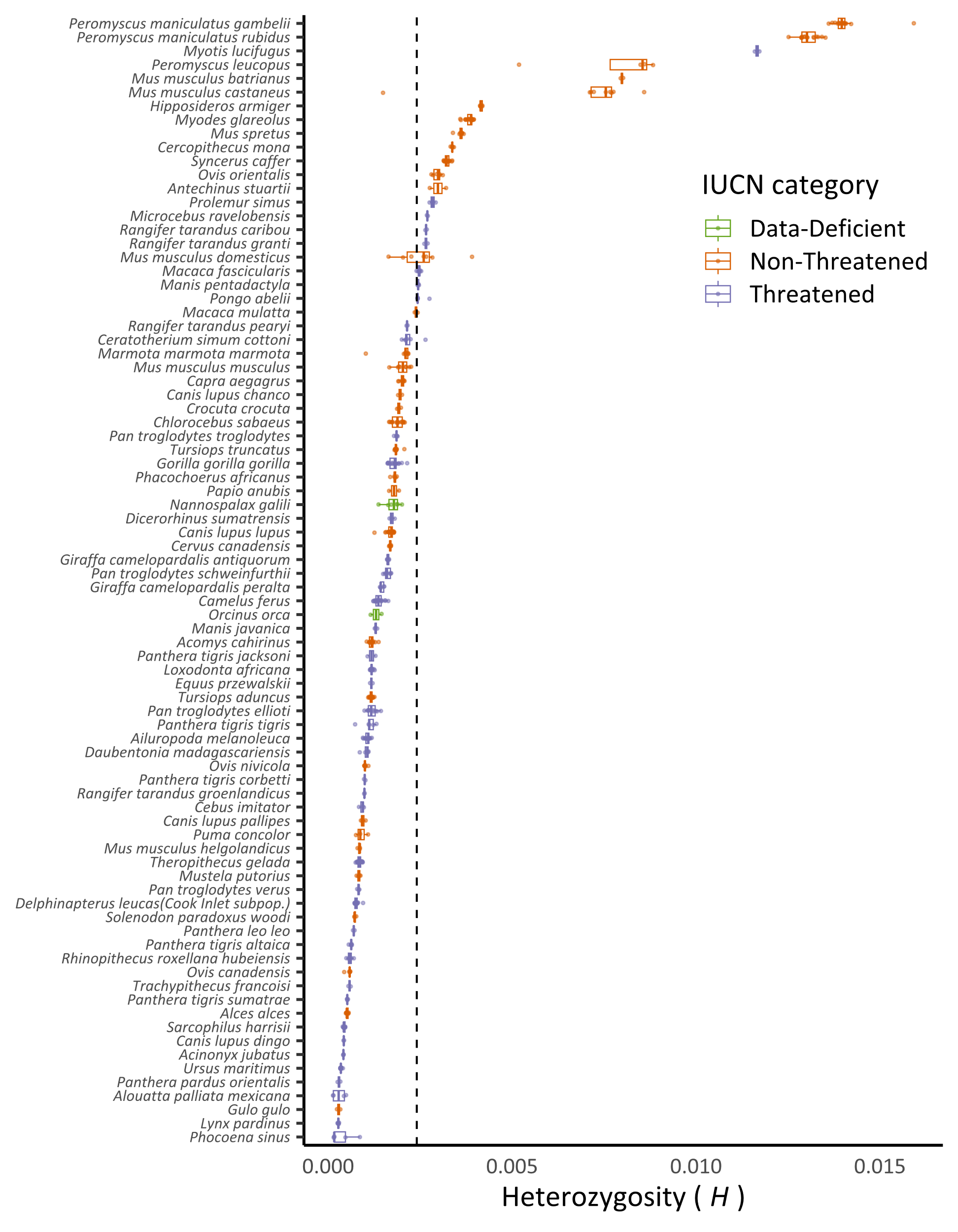
Fig. S8. A box plot of heterozygosity by species. Species are arranged by descending median value of heterozygosity and colored by IUCN Threatened/Non-Threatened categories, plus “Data-Deficient”. The boxes represent the range between the first and the third quartile (interquartile range, IQR) with the median line inside. The whiskers above and below the box represent the largest and smallest values within 1.5 IQR, respectively. Dashed line indicates the overall mean value. Species names (according to NCBI) are shown on the y-axis.


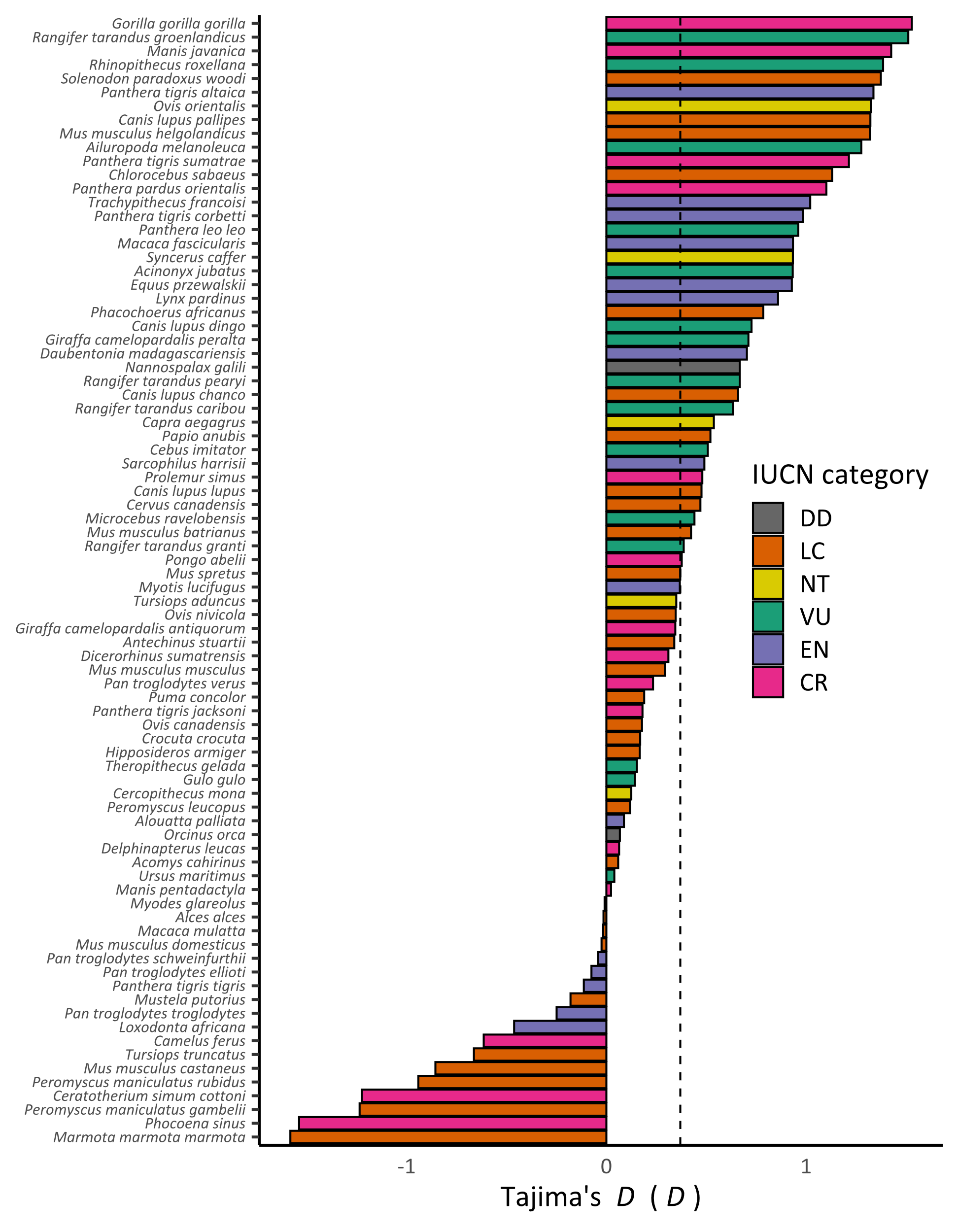
Fig. S9. A bar plot of Tajima’s *D* by species. Species are arranged by descending value of Tajima’s *D* and colored by IUCN full categories. Dashed line indicates the overall mean value. Species names (according to NCBI) are shown on the y-axis.


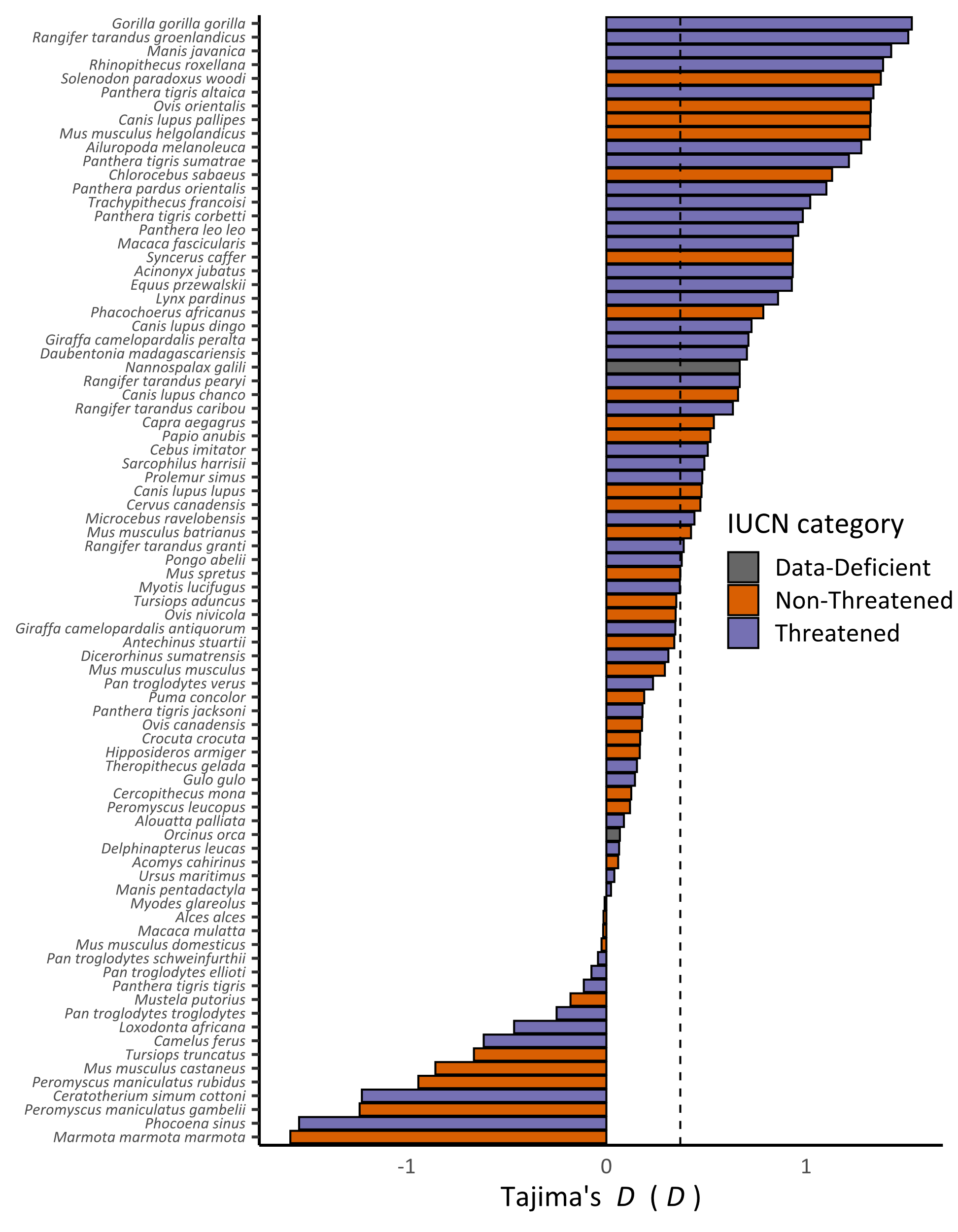
Fig. S10. A bar plot of Tajima’s *D* by species. Species are arranged by descending value of Tajima’s *D* and colored by IUCN Threatened/Non-Threatened categories, plus “Data-Deficient”. Dashed line indicates the overall mean value. Species names (according to NCBI) are shown on the y-axis.


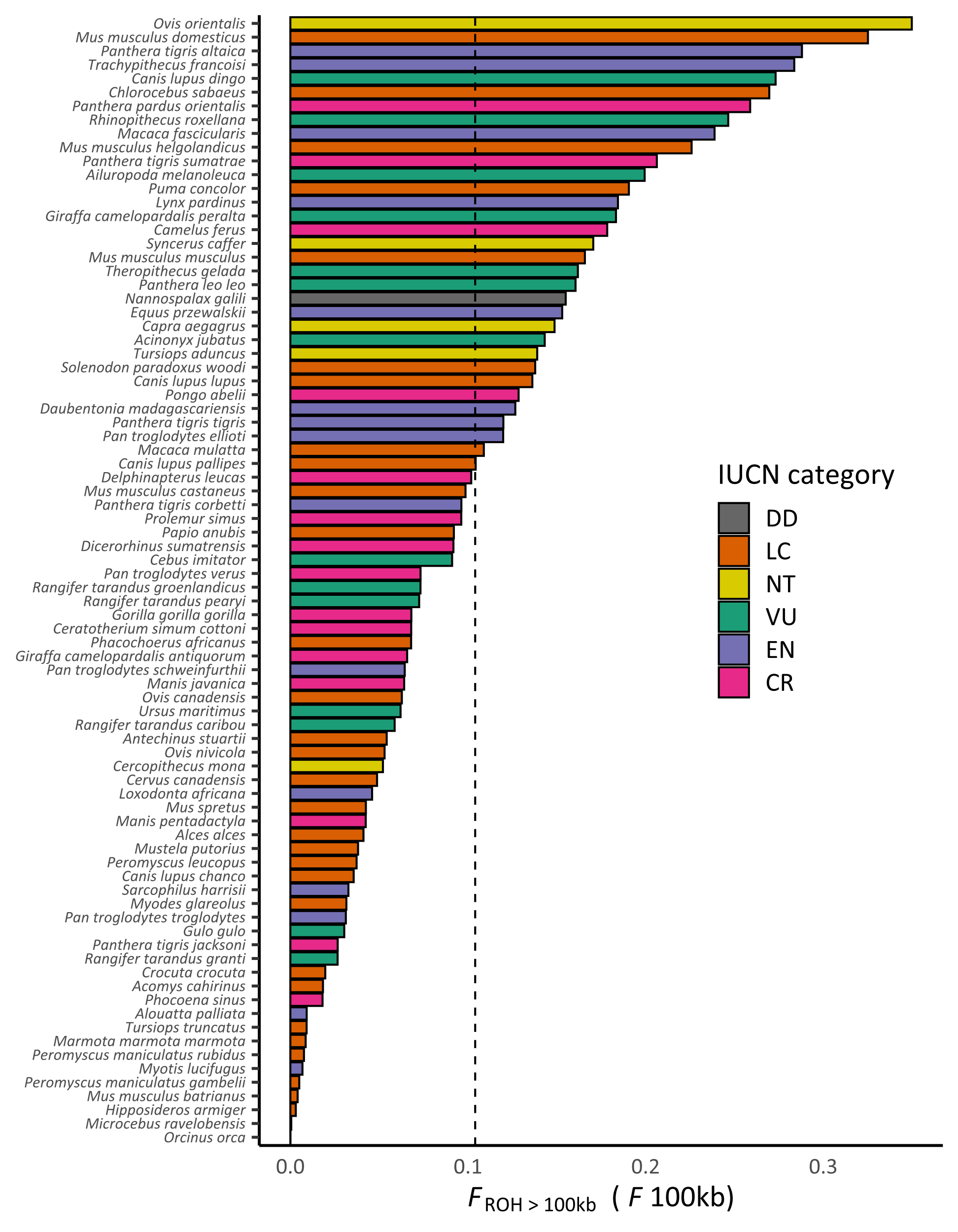
Fig. S11. A bar plot of *F*_ROH > 100kb_ by species. Species are arranged by descending value of *F*_ROH > 100kb_ and colored by IUCN full categories. Dashed line indicates the overall mean value. Variance is high but on average, ~10% of the mammalian genomes are autozygous in our dataset. Species names (according to NCBI) are shown on the y-axis.


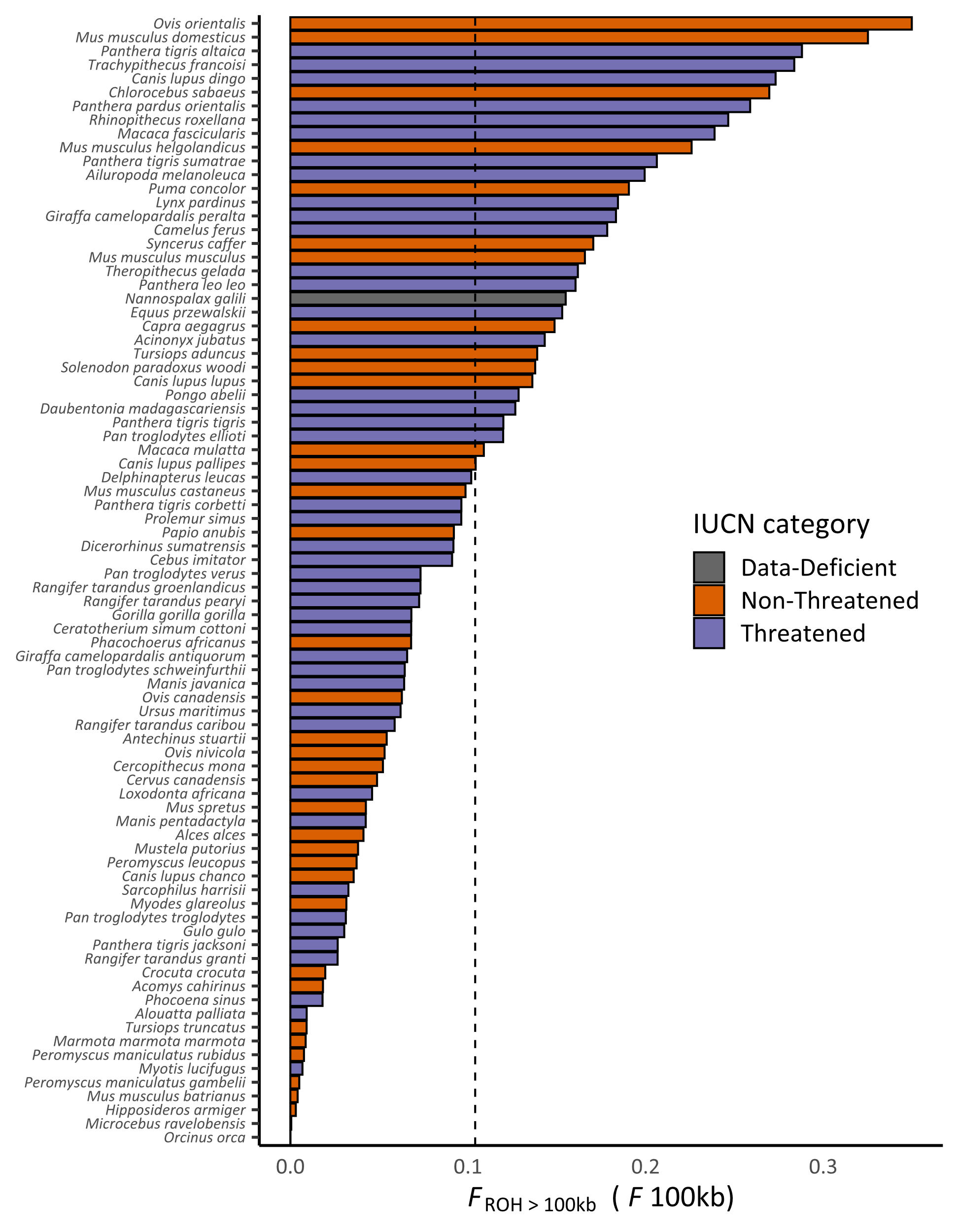


**Fig. S12.** A bar plot of *F*_ROH > 100kb_ by species. Species are arranged by descending value of *F*_ROH > 100kb_ and colored by IUCN Threatened/Non-Threatened categories, plus “Data-Deficient”. Dashed line indicates the overall mean value. Variance is high but on average, ~10% of the mammalian genomes are autozygous in our dataset. Species names (according to NCBI) are shown on the y-axis.

**
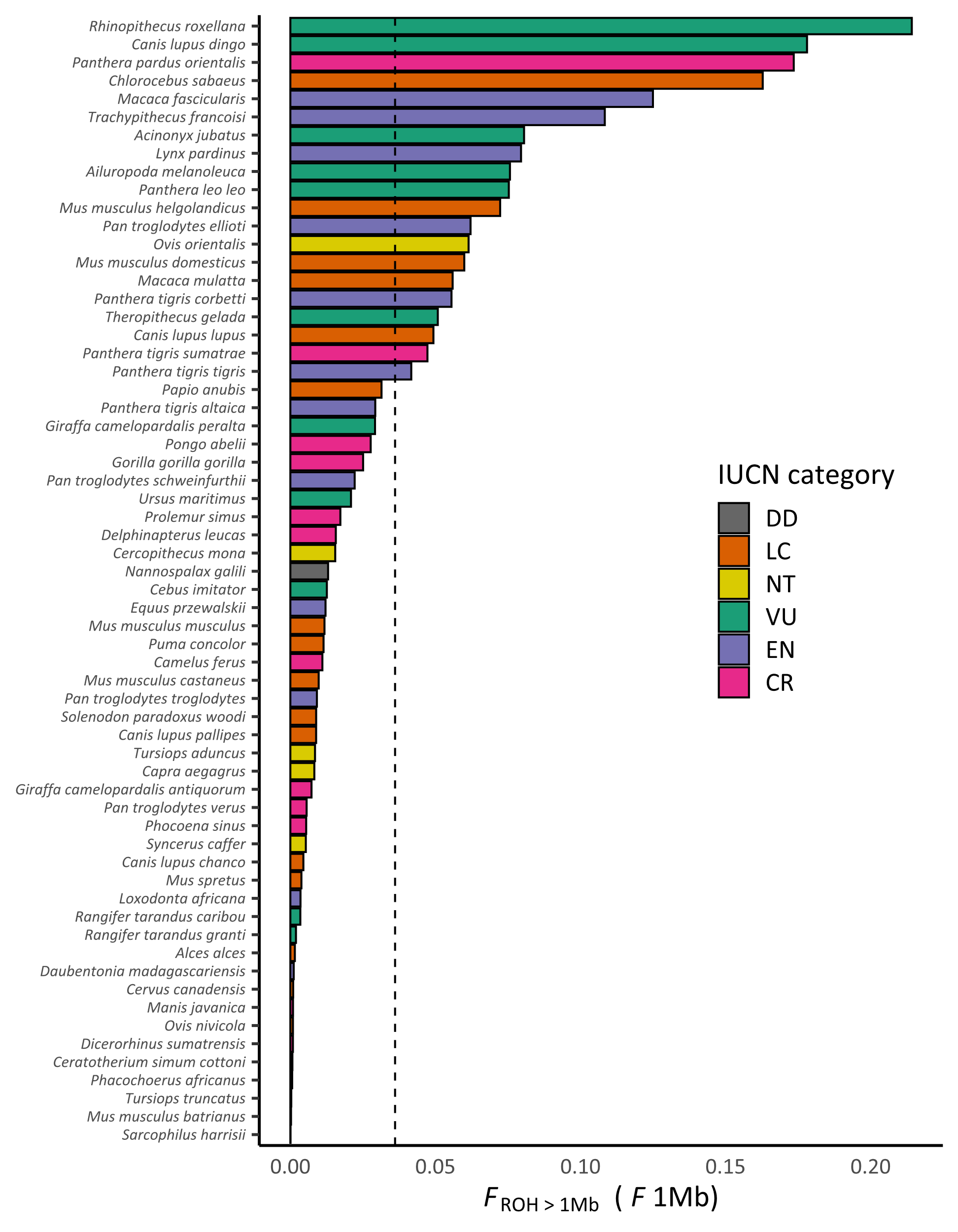
Fig. S13.** A bar plot of *F*_ROH > 1Mb_ by species. Species are arranged by descending value of *F*_ROH > 1Mb_ and colored by IUCN full categories. Dashed line indicates the overall mean value. Species names (according to NCBI) are shown on the y-axis.


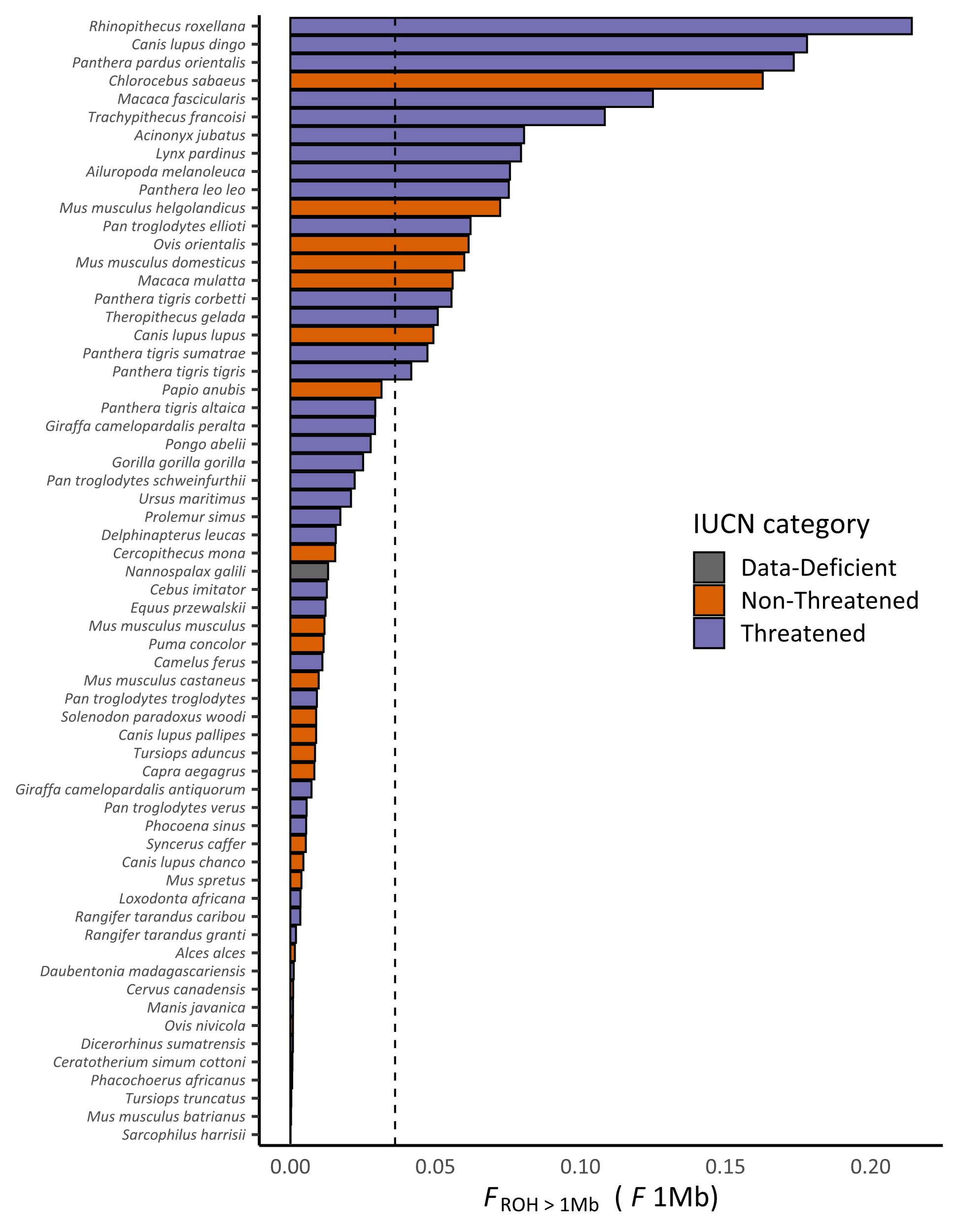


**Fig. S14.** A bar plot of *F*_ROH > 1Mb_ by species. Species are arranged by descending value of *F*_ROH > 1Mb_ and colored by IUCN Threatened/Non-Threatened categories, plus “Data-Deficient”. Dashed line indicates the overall mean value. Species names (according to NCBI) are shown on the y-axis.


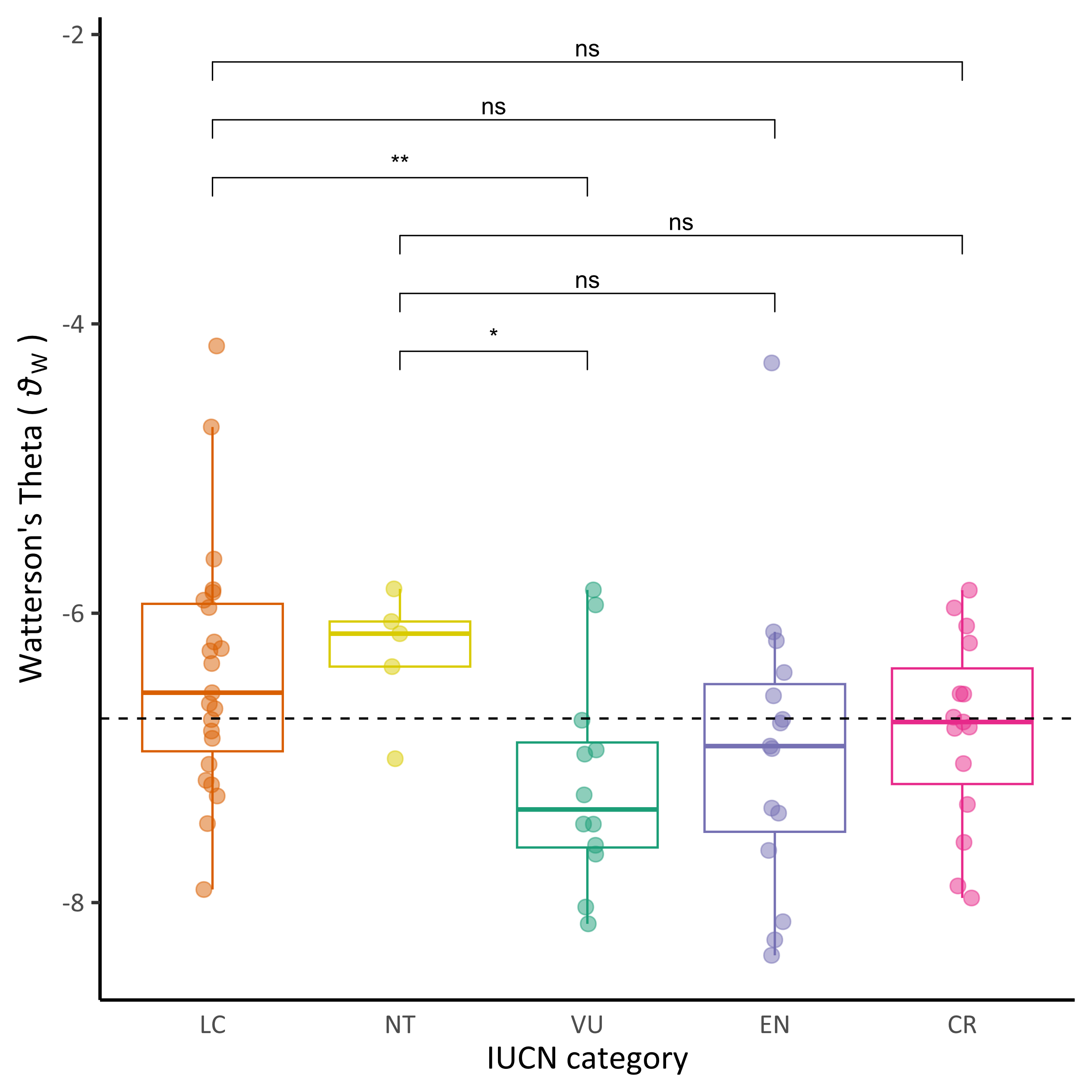


**Fig. S15.** A box plot of log-transformed heterozygosity against IUCN full categories. Statistical difference among IUCN categories was assessed using a Kruskal-Wallis test (*p* = 0.0038). Each Non-Threatened category (i.e., “LC” and “NT”) is compared to each Threatened category (i.e., “VU”, “EN”, and “CR”) and the significance is shown (ns: non-significant, *: *p* < 0.05, **: *p* < 0.01). The boxes represent the range between the first and the third quartile (interquartile range, IQR) with the median line inside. The whiskers above and below the box represent the largest and smallest values within 1.5 IQR, respectively. Dashed line indicates the overall mean value.


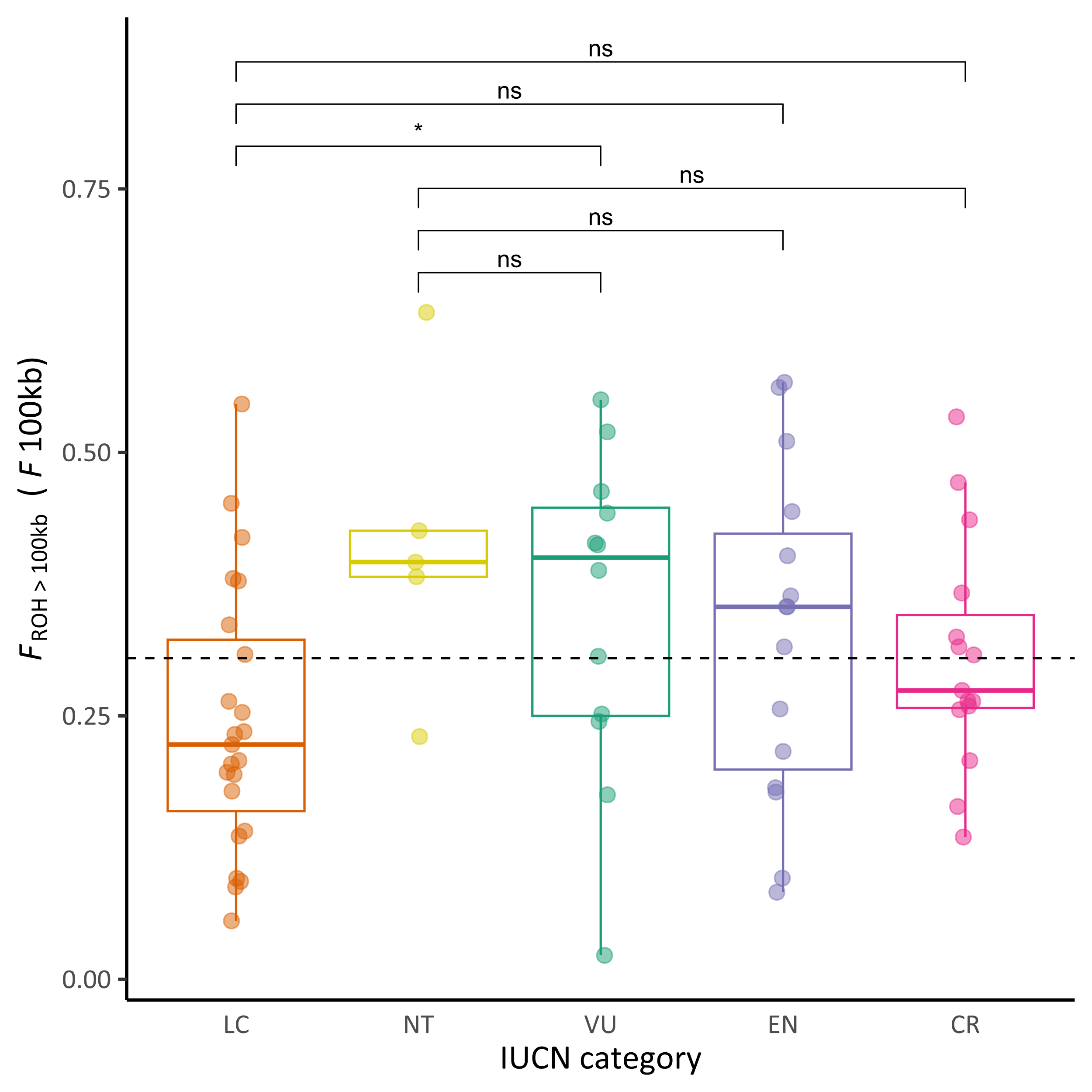
Fig. S16. A box plot of arcsine-transformed *F*_ROH > 100kb_ against IUCN full categories. Statistical difference among IUCN categories was assessed using a Kruskal-Wallis test (*p* = 0.044). Each non-threatened category (i.e., “LC” and “NT”) is compared to each threatened category (i.e., “VU”, “EN”, and “CR”) and the significance is shown (ns: non-significant, *: *p* < 0.05). The boxes represent the range between the first and the third quartile (interquartile range, IQR) with the median line inside. The whiskers above and below the box represent the largest and smallest values within 1.5 IQR, respectively. Dashed line indicates the overall mean value.


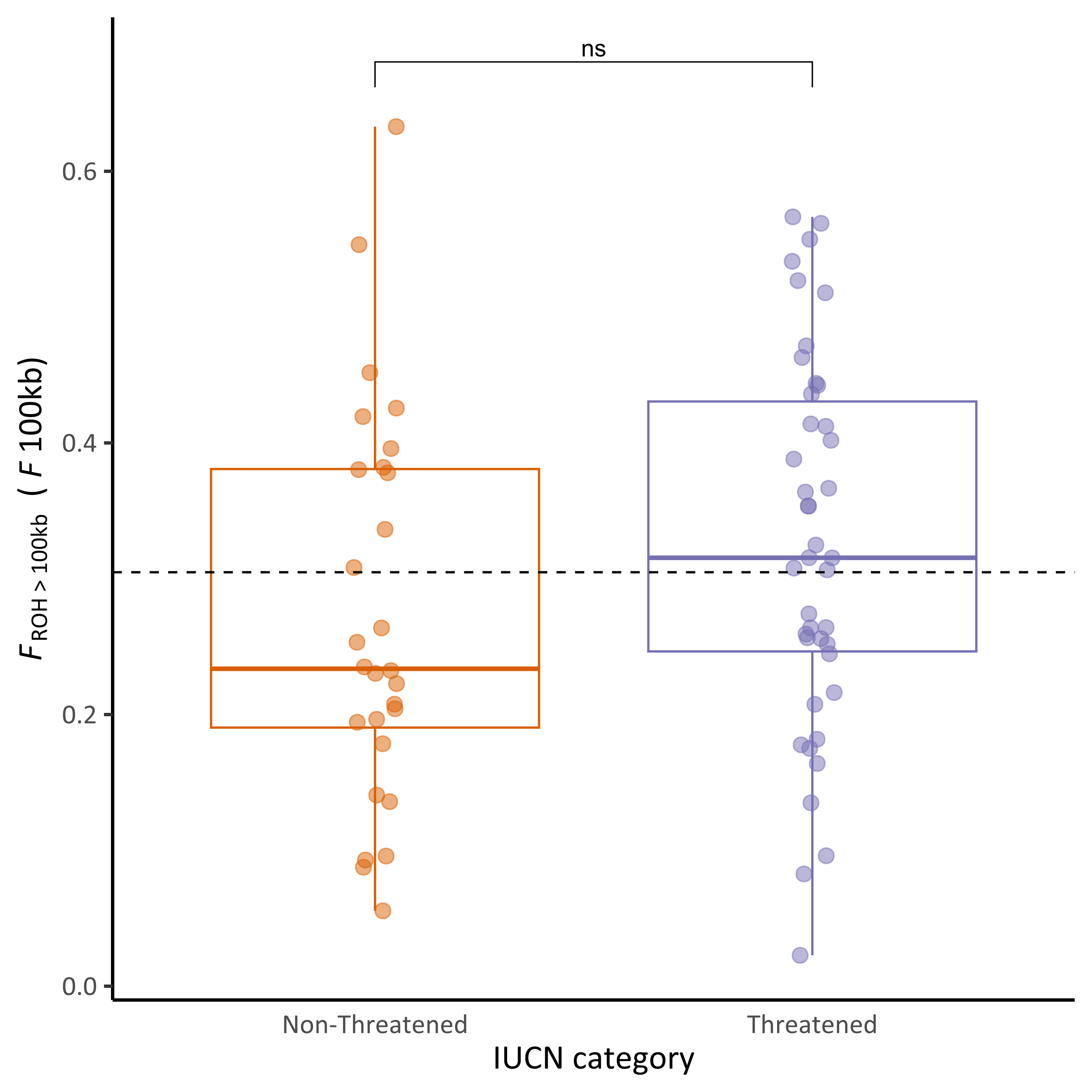


**Fig. S17.** A box plot of arcsine-transformed *F*_ROH > 100kb_ against IUCN Threatened/Non-Threatened categories. Non-Threatened category is compared to Threatened category using a Wilcoxon test and the significance is shown (ns: non-significant). The boxes represent the range between the first and the third quartile (interquartile range, IQR) with the median line inside. The whiskers above and below the box represent the largest and smallest values within 1.5 IQR, respectively. Dashed line indicates the overall mean value.


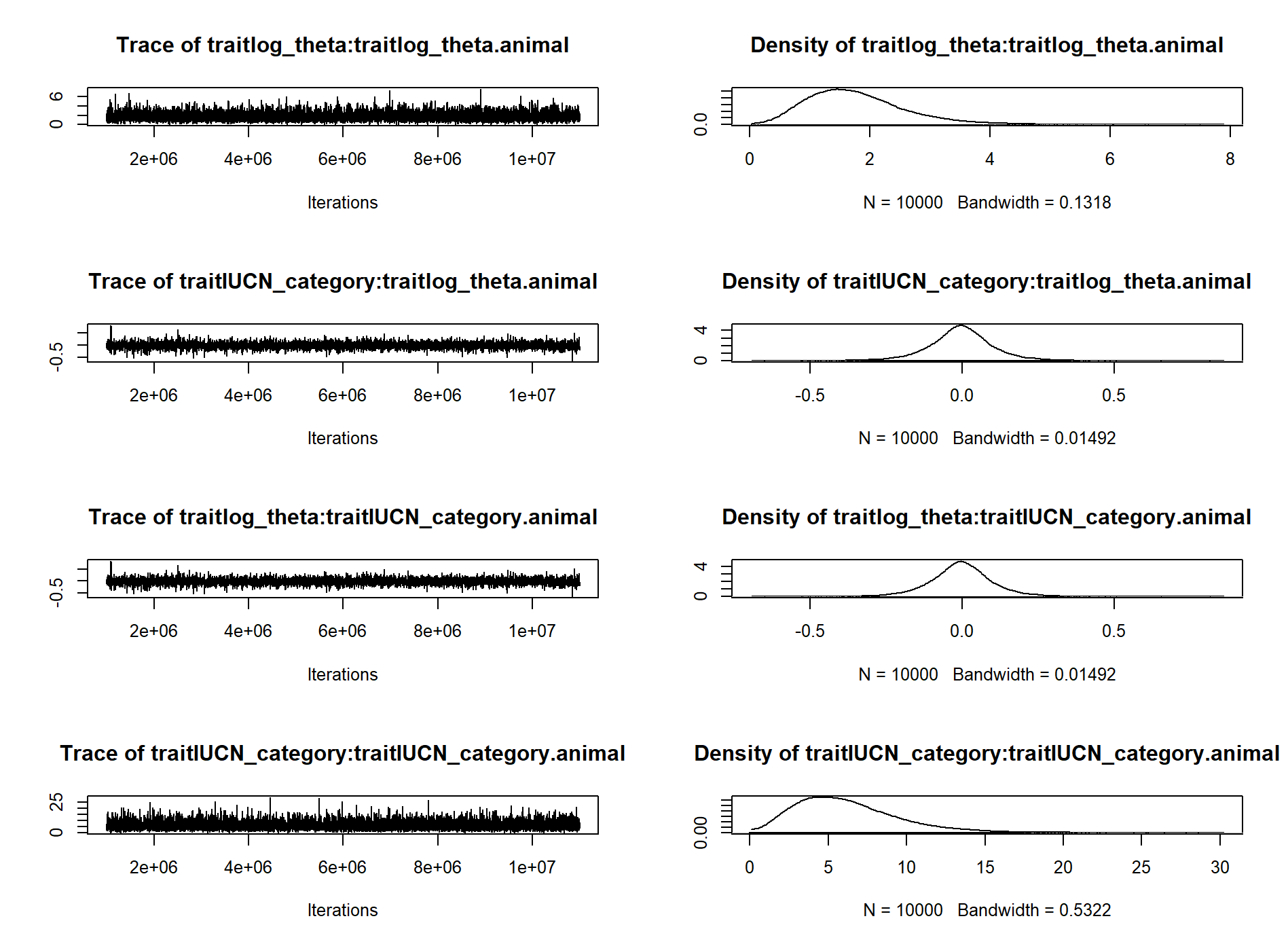

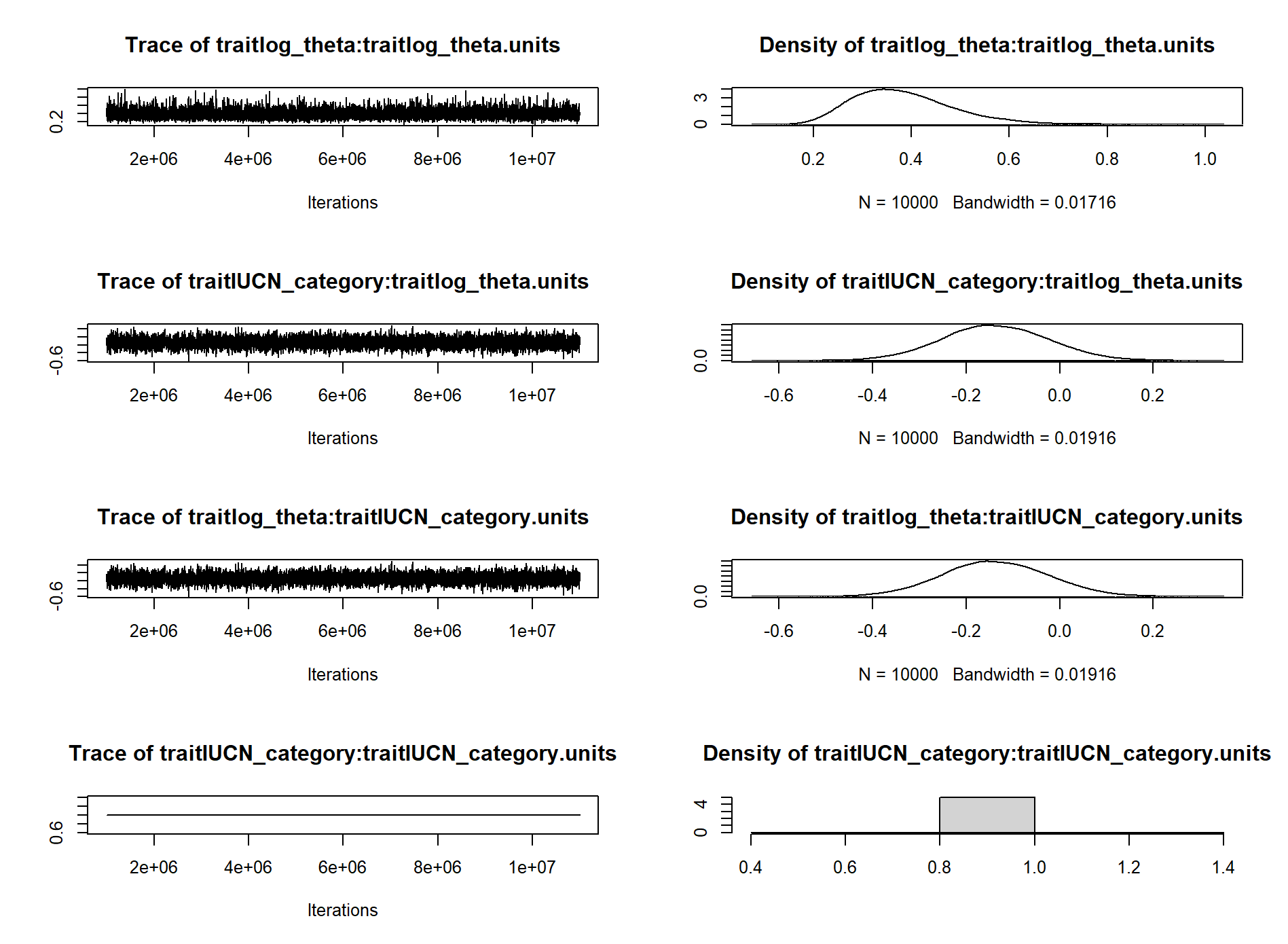


Fig. S18. MCMC (Markov Chain Monte Carlo) trace plots of the multi-response phylogenetic mixed model of “cbind(log(*θ*_W_), IUCN full category) ~ trait-1” from ‘MCMCglmm’. All traces converged. The trace of “trait:trait.units” which is shown at the bottom left was fixed to 1 in the prior setting.


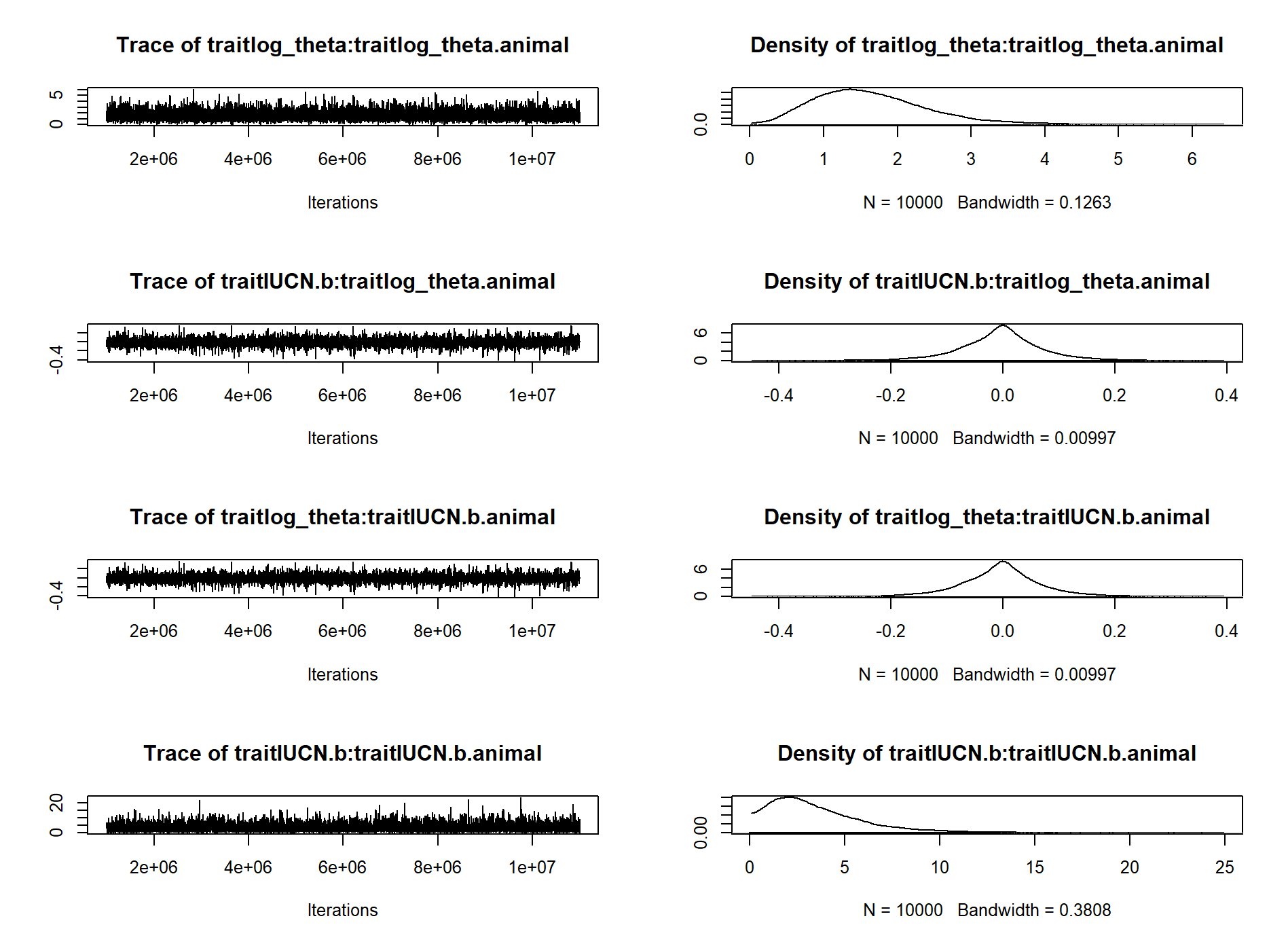

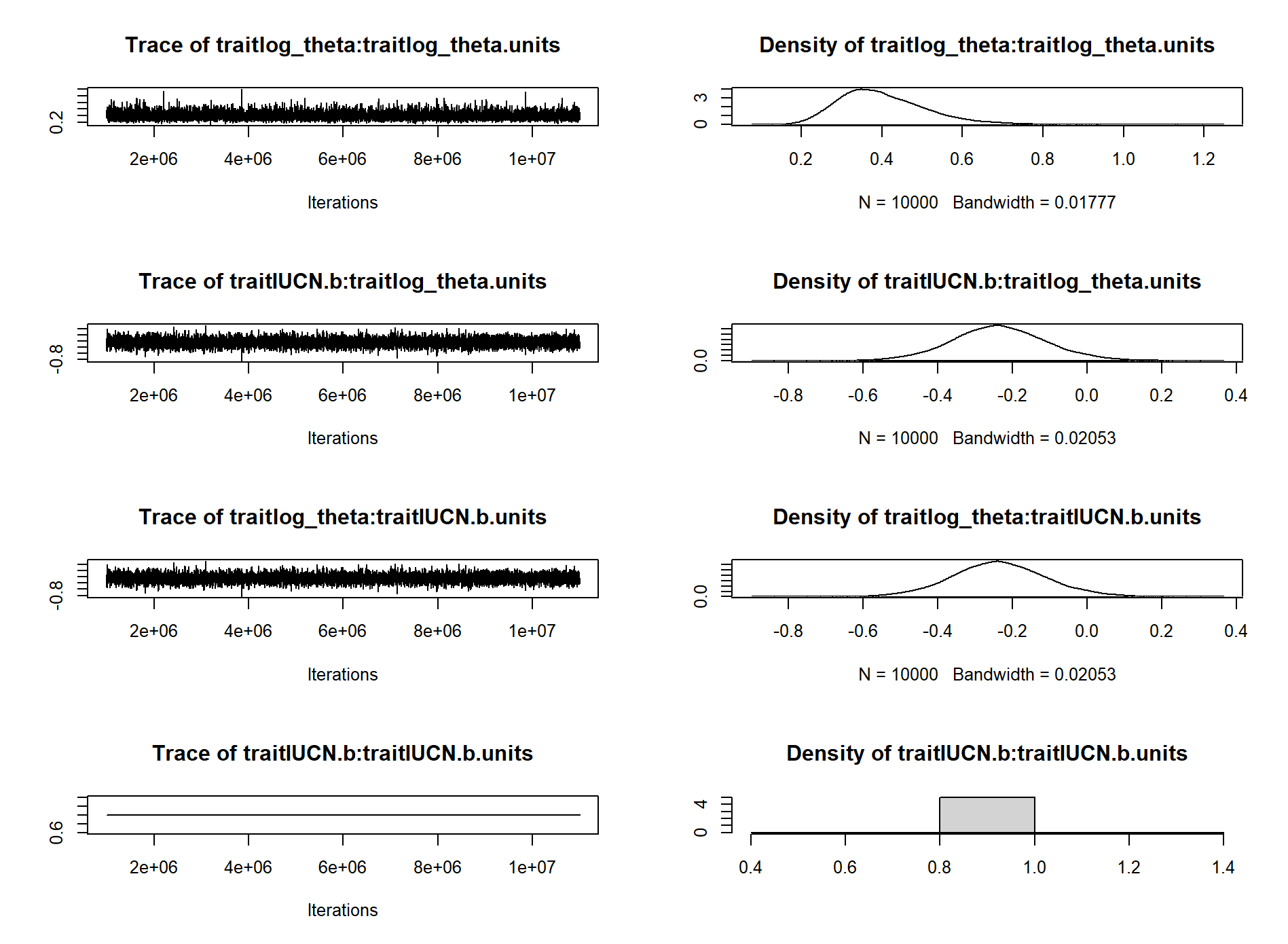


Fig. S19. MCMC (Markov Chain Monte Carlo) trace plots of the multi-response phylogenetic mixed model of “cbind(log(*θ*_W_), IUCN binary category) ~ trait-1” from ‘MCMCglmm’. All traces converged. The trace of “trait:trait.units” which is shown at the bottom left was fixed to 1 in the prior setting.


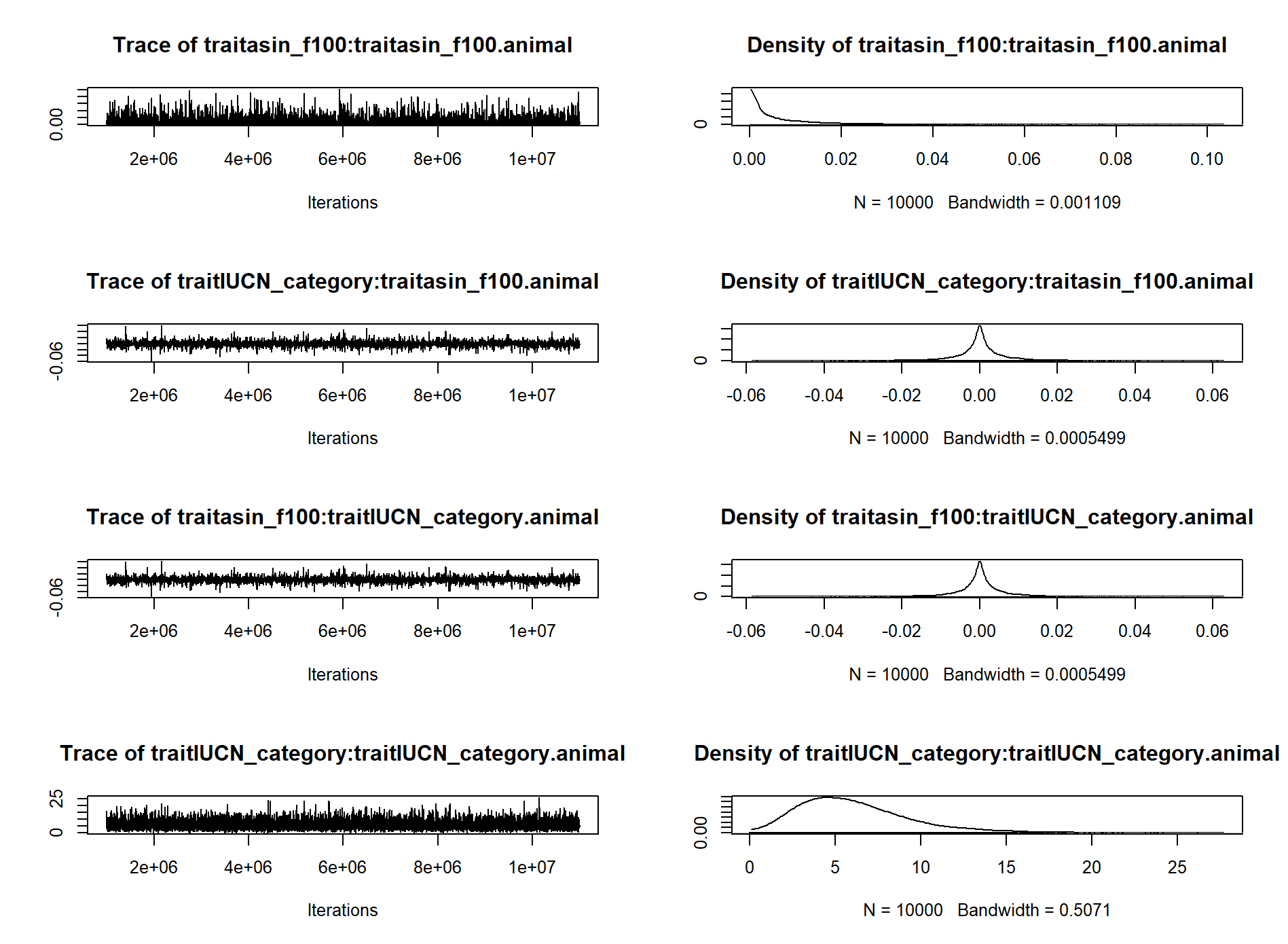

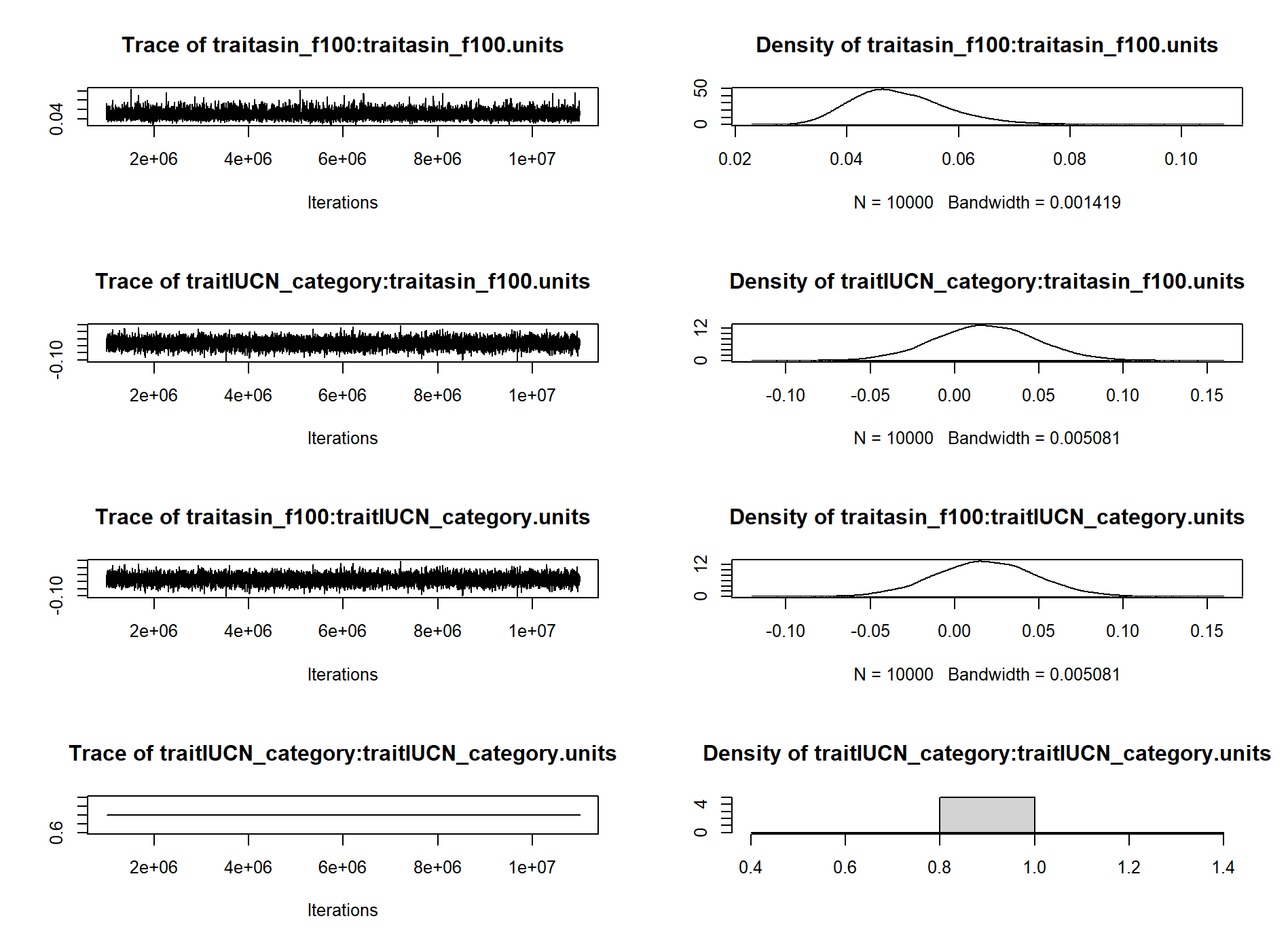


Fig. S20. MCMC (Markov Chain Monte Carlo) trace plots of the multi-response phylogenetic mixed model of “cbind(asin(sqrt((*F*100kb)), IUCN full category) ~ trait-1” from ‘MCMCglmm’. All traces converged. The trace of “trait:trait.units” which is shown at the bottom left was fixed to 1 in the prior setting.


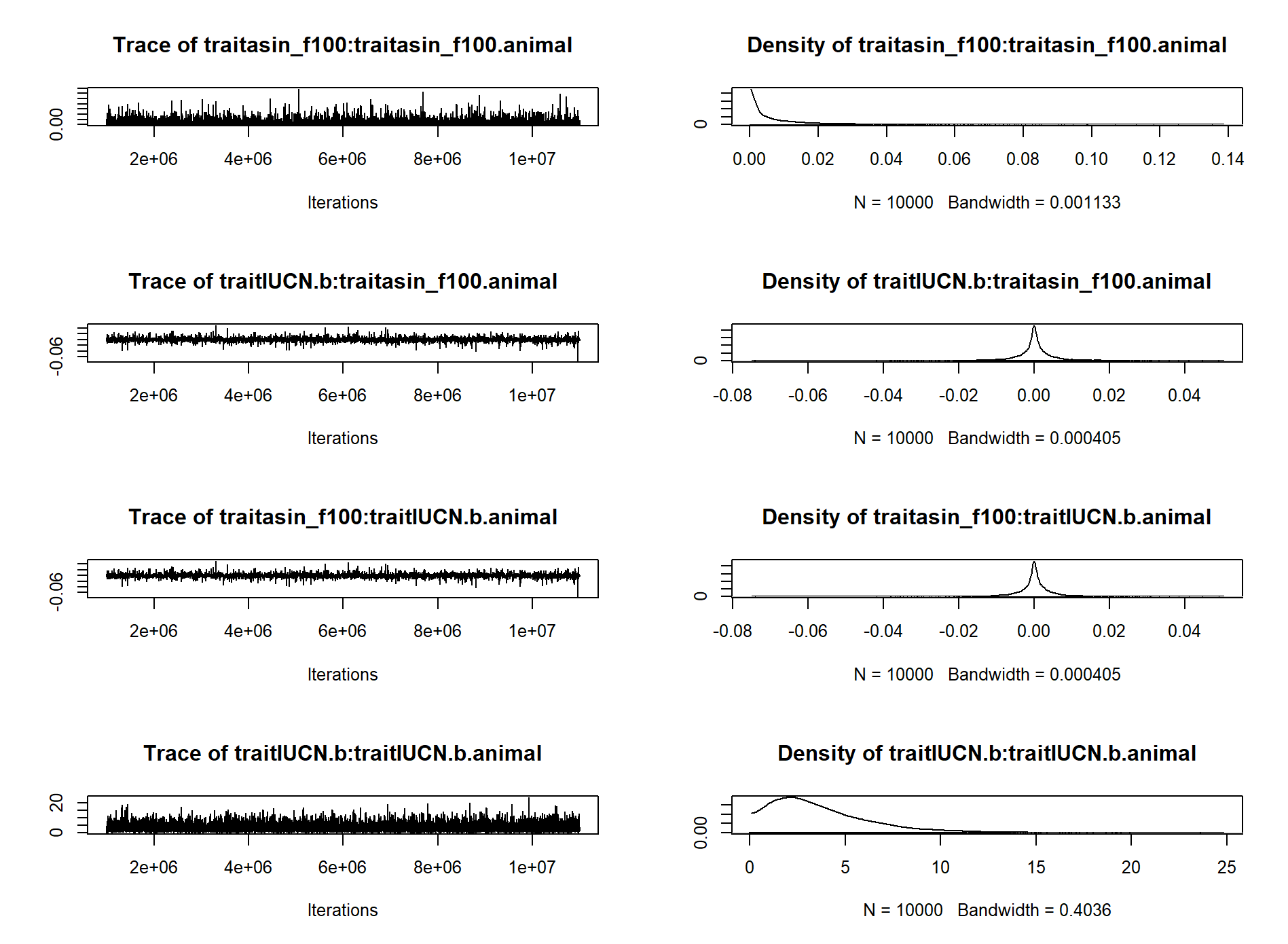

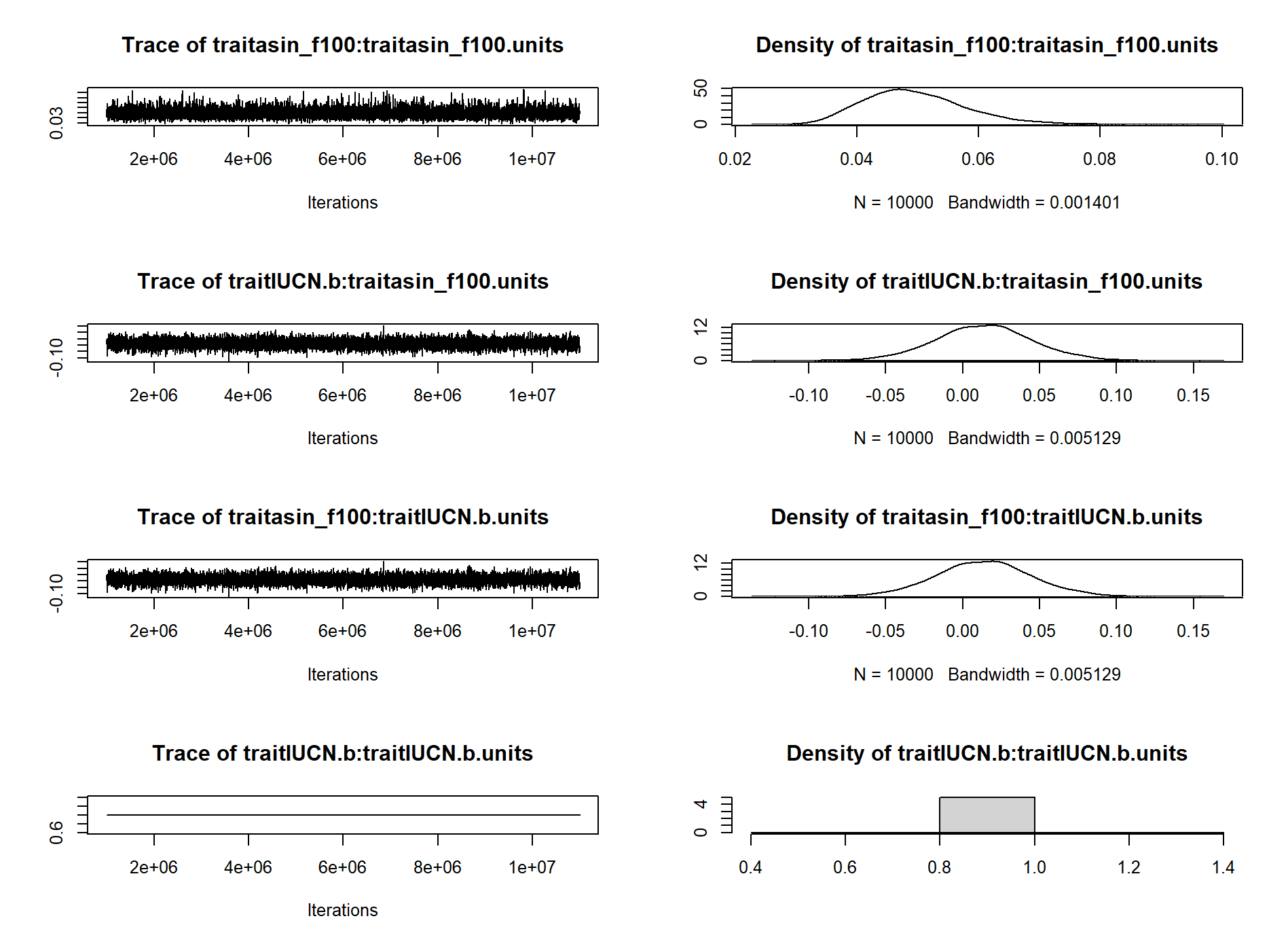


Fig. S21. MCMC (Markov Chain Monte Carlo) trace plots of the multi-response phylogenetic mixed model of “cbind(asin(sqrt((*F*100kb)), IUCN binary category) ~ trait-1” from ‘MCMCglmm’. All traces converged. The trace of “trait:trait.units” which is shown at the bottom left was fixed to 1 in the prior setting.

Table S1. Genomic diversity metrics grouped by taxonomic Orders. Abbreviations: spp. No. = the number of species, *θ*_W_ = Watterson’s Theta, *H* = observed genome-wide heterozygosity, 𝜋 = nucleotide diversity, *D* = Tajima’s *D*, *F*100kb = *F*_ROH >100kb_, *F*1Mb = *F*_ROH >1Mb_, NA = not applicable.

| Order | spp.  No. | mean  (*θ*_W_) | sd  (*θ*_W_) | mean  (*H*) | sd  (*H*) | mean  (𝜋) | sd  (𝜋) | mean  (*D*) | sd  (*D*) | mean  (*F*100kb) | sd  (*F*100kb) |
| --- | --- | --- | --- | --- | --- | --- | --- | --- | --- | --- | --- |
| Artiodactyla | 15 | 0.00151 | 0.00072 | 0.00178 | 0.00084 | 0.00170 | 0.00084 | 0.54710 | 0.51422 | 0.10675 | 0.08560 |
| Carnivora | 19 | 0.00090 | 0.00056 | 0.00088 | 0.00053 | 0.00101 | 0.00060 | 0.64643 | 0.51476 | 0.13545 | 0.08710 |
| Cetacea | 5 | 0.00102 | 0.00060 | 0.00109 | 0.00056 | 0.00098 | 0.00054 | -0.34401 | 0.76587 | 0.05363 | 0.06267 |
| Chiroptera | 2 | 0.00880 | 0.00734 | 0.00791 | 0.00531 | 0.00918 | 0.00760 | 0.26754 | 0.14183 | 0.00495 | 0.00265 |
| Dasyuromorphia | 2 | 0.00158 | 0.00190 | 0.00171 | 0.00180 | 0.00165 | 0.00190 | 0.41532 | 0.10654 | 0.04346 | 0.01523 |
| Eulipotyphla | 1 | 0.00104 | NA | 0.00073 | NA | 0.00127 | NA | 1.37423 | NA | 0.13783 | NA |
| Perissodactyla | 3 | 0.00162 | 0.00058 | 0.00170 | 0.00052 | 0.00152 | 0.00025 | 0.00503 | 1.10849 | 0.10429 | 0.04385 |
| Pholidota | 2 | 0.00161 | 0.00057 | 0.00187 | 0.00082 | 0.00179 | 0.00042 | 0.72478 | 0.99199 | 0.05326 | 0.01529 |
| Primates | 19 | 0.00157 | 0.00092 | 0.00166 | 0.00089 | 0.00171 | 0.00094 | 0.48682 | 0.51163 | 0.11907 | 0.08512 |
| Proboscidea | 1 | 0.00097 | NA | 0.00118 | NA | 0.00092 | NA | -0.46186 | NA | 0.04602 | NA |
| Rodentia | 13 | 0.00626 | 0.00643 | 0.00520 | 0.00444 | 0.00543 | 0.00475 | -0.10738 | 0.82196 | 0.08657 | 0.10215 |
|  |  |  |  |  |  |  |  |  |  |  |  |
| Order | spp.  No. | mean  (*F*1Mb) | sd  (*F*1Mb) | DD | LC | NT | VU | EN | CR | Non-  Threatened | Threatened |
| Artiodactyla | 15 | 0.01100 | 0.01775 | 0 | 5 | 3 | 5 | 0 | 2 | 8 | 7 |
| Carnivora | 19 | 0.06209 | 0.05291 | 0 | 6 | 0 | 6 | 4 | 3 | 6 | 13 |
| Cetacea | 5 | 0.00749 | 0.00642 | 1 | 1 | 1 | 0 | 0 | 2 | 2 | 2 |
| Chiroptera | 2 | NA | NA | 0 | 1 | 0 | 0 | 1 | 0 | 1 | 1 |
| Dasyuromorphia | 2 | 0.00007 | NA | 0 | 1 | 0 | 0 | 1 | 0 | 1 | 1 |
| Eulipotyphla | 1 | 0.00889 | NA | 0 | 1 | 0 | 0 | 0 | 0 | 1 | 0 |
| Perissodactyla | 3 | 0.00457 | 0.00654 | 0 | 0 | 0 | 0 | 1 | 2 | 0 | 3 |
| Pholidota | 2 | 0.00092 | NA | 0 | 0 | 0 | 0 | 0 | 2 | 0 | 2 |
| Primates | 19 | 0.05569 | 0.06138 | 0 | 3 | 1 | 4 | 7 | 4 | 4 | 15 |
| Proboscidea | 1 | 0.00349 | NA | 0 | 0 | 0 | 0 | 1 | 0 | 0 | 1 |
| Rodentia | 13 | 0.02441 | 0.02907 | 1 | 12 | 0 | 0 | 0 | 0 | 12 | 0 |

**Table S2.** Phylogenetic Generalized Least Squares model results from ‘caper’ and ‘sjstats’. The model formula represents ‘dependent variable ~ independent variable’ excluding technical factors of independent variables. Significant *p*-values are bolded and their associated effect size values are italicized. Abbreviations: *θ*_W_ = Watterson’s Theta, *D* = Tajima’s *D*, *F*100kb = *F*_ROH > 100kb_, *F*1Mb = *F*_ROH > 1Mb_, df = degrees of freedom, Indep. *p* = *p*-value of the independent variable as an individual factor in the model. Indep. eff. = effect size (partial *ω*^2^) of the independent variable as an individual factor in the model.

| Model formula | df | *F*-statistic | *p*-value | Adjusted-*R*^2^ | 𝜆 (95% CI) | Indep. *p* | Indep. eff. |
| --- | --- | --- | --- | --- | --- | --- | --- |
| log(*θ*_W_) ~ IUCN full category | 8, 61 | 2.469 | **0.022** | *0.146* | 0.961  (0.883–0.988) | **0.028** | *0.099* |
| log(*θ*_W_) ~ IUCN binary category | 5, 64 | 3.194 | **0.012** | *0.137* | 0.951  (0.857–0.983) | **0.003** | *0.106* |
| *D* ~ IUCN full category | 8, 61 | 1.857 | 0.084 | 0.090 | 0  (NA–0.575) | 0.162 | 0.038 |
| *D* ~ IUCN binary category | 5, 64 | 1.768 | 0.132 | 0.053 | 0  (NA–0.767) | 0.178 | 0.012 |
| asin(sqrt(*F*100kb)) ~ IUCN full category | 8, 61 | 3.166 | **0.005** | *0.201* | 0  (NA–0.649) | **0.022** | *0.106* |
| asin(sqrt(*F*100kb)) ~ IUCN binary category | 5, 64 | 3.523 | **0.007** | *0.155* | 0  (NA–0.715) | 0.304 | 0.001 |
| asin(sqrt(*F*1Mb)) ~ IUCN full category | 8, 45 | 1.103 | 0.379 | 0.015 | 0.716  (0.219–0.930) | 0.213 | 0.037 |
| asin(sqrt(*F*1Mb)) ~ IUCN binary category | 5, 48 | 1.183 | 0.331 | 0.017 | 0.799  (0.348–0.949) | 0.112 | 0.029 |
| log(*θ*_W_) ~ population trend | 7, 62 | 2.013 | 0.068 | 0.093 | 0.958  (0.872–0.986) | 0.117 | 0.043 |
| *D* ~ population trend | 7, 62 | 1.727 | 0.119 | 0.069 | 0  (NA–0.781) | 0.228 | 0.020 |
| asin(sqrt(*F*100kb)) ~ population trend | 7, 62 | 2.896 | **0.011** | *0.161* | 0  (NA–0.647) | 0.172 | 0.030 |
| asin(sqrt(*F*1Mb)) ~ population trend | 7, 46 | 0.937 | 0.488 | -0.008 | 0.787  (0.380–0.942) | 0.321 | 0.011 |
| log(*θ*_W_) ~ log(geographic range) | 4, 65 | -9.871 | 1 | -1.704 | 0.995  (0.983–0.998) | 0.088 | 0.027 |
| *D* ~ log(geographic range) | 4, 65 | 1.254 | 0.297 | 0.014 | 0  (NA–0.699) | 0.161 | 0.014 |
| asin(sqrt(*F*100kb)) ~ log(geographic range) | 4, 65 | -2.122 | 1 | -0.221 | 0.575  (0.026–0.865) | 0.174 | 0.0122 |
| asin(sqrt(*F*1Mb)) ~ log(geographic range) | 4, 49 | 1.027 | 0.403 | 0.002 | 0.869  (0.584–0.968) | **0.006** | *0.115* |
| IUCN full category ~ log(*θ*_W_) | 4, 65 | 2.259 | 0.072 | 0.068 | 0.930  (0.738–0.983) | **0.019** | 0.063 |
| IUCN binary category ~ log(*θ*_W_) | 4, 65 | 3.814 | **0.008** | *0.140* | 0.879  (NA–0.976) | **0.002** | *0.121* |
| IUCN full category ~ asin(sqrt(*F*100kb)) | 4, 65 | 0.273 | 0.894 | -0.044 | 0.964  (0.892–0.990) | 0.187 | 0.011 |
| IUCN binary category ~ asin(sqrt(*F*100kb)) | 4, 65 | -2.660 | 1 | -0.269 | 1  (0.999–NA) | 0.544 | -0.009 |
| log(*θ*_W_) ~ trophic level | 6, 56 | 1.795 | 0.117 | 0.071 | 0.972  (0.890–0.998) | 0.427 | -0.004 |
| *D* ~ trophic level | 6, 56 | 1.272 | 0.285 | 0.026 | 0  (NA–0.729) | 0.677 | -0.020 |
| asin(sqrt(*F*100kb)) ~ trophic level | 6, 56 | 2.433 | **0.037** | *0.122* | 0  (NA–0.676) | 0.133 | 0.034 |
| asin(sqrt(*F*1Mb)) ~ trophic level | 6, 40 | 0.668 | 0.676 | -0.045 | 0.697  (0.251–0.903) | 0.672 | -0.026 |
| log(*θ*_W_) ~ log(body mass) | 4, 58 | -7.432 | 1 | -1.193 | 0.995  (0.966–NA) | 0.529 | -0.009 |
| *D* ~ log(body mass) | 4, 58 | 0.604 | 0.661 | -0.026 | 0  (NA–0.744) | 0.994 | -0.016 |
| asin(sqrt(*F*100kb)) ~ log(body mass) | 4, 58 | -0.749 | 1 | -0.127 | 0.606  (0.121–0.877) | 0.250 | 0.005 |
| asin(sqrt(*F*1Mb)) ~ log(body mass) | 4, 42 | 0.506 | 0.731 | -0.045 | 0.725  (0.359–0.908) | 0.795 | -0.020 |
| log(*θ*_W_) ~ sqrt(habitat breadth) | 4, 41 | -5.437 | 1 | -1.337 | 1  (0.992–NA) | 0.903 | -0.021 |
| *D* ~ sqrt(habitat breadth) | 4, 41 | 1.718 | 0.165 | 0.060 | 0  (NA–0.639) | 0.929 | -0.022 |
| asin(sqrt(*F*100kb)) ~ sqrt(habitat breadth) | 4, 41 | -0.589 | 1 | -0.164 | 0.381  (NA–0.802) | 0.452 | -0.009 |
| asin(sqrt(*F*1Mb)) ~ sqrt(habitat breadth) | 4, 41 | 0.927 | 0.458 | -0.007 | 0.655  (0.209–0.889) | 0.575 | -0.015 |

**Table S3.** The result of comparisons among significant Phylogenetic Generalized Least Squares models. The models were compared by Akaike Information Criterion (AIC) and arranged by ascending order of AIC. Abbreviations: *θ*_W_ = Watterson’s Theta, *F*100kb = *F*_ROH > 100kb_

| Model | degrees of freedom | AIC |
| --- | --- | --- |
| asin(sqrt(*F*100kb)) ~ IUCN binary category | 6 | -77.146 |
| asin(sqrt(*F*100kb)) ~ IUCN full category | 9 | -78.437 |
| log(*θ*_W_) ~ IUCN binary category | 6 | 165.510 |
| log(*θ*_W_) ~ IUCN full category | 9 | 167.635 |

**Table S4.** The result of Multi-Response Phylogenetic Mixed Models with a GD and IUCN categorization as response variables. Only significant GD metrics identified from the Phylogenetic Generalized Least Squares models were tested. Values of Deviance Information Criterion (DIC), independent correlation (link-scale, median), phylogenetic correlation (link-scale, median), and phylogenetic signal (link-scale, median) are shown. Abbreviations: *H* = Heterozygosity, *F*100kb = *F*_ROH > 100kb_

| Model | DIC | Independent  correlation | Phylogenetic  correlation | Phylogenetic signal  of GD | Phylogenetic signal  of Categorization |
| --- | --- | --- | --- | --- | --- |
| cbind(log(*θ*_W_), IUCN full category) ~ trait-1 | 327.996 | -0.234 | -0.002 | 0.548 | 0.739 |
| cbind(log(*θ*_W_), IUCN binary category) ~ trait-1 | 225.594 | -0.391 | -0.002 | 0.527 | 0.592 |
| cbind(asin(sqrt(*F*100kb)), IUCN full category) ~ trait-1 | 135.512 | 0.082 | 0.000 | 0.003 | 0.736 |
| cbind(asin(sqrt(*F*100kb)), IUCN binary category) ~ trait-1 | 31.583 | 0.063 | 0.001 | 0.003 | 0.605 |

**Table S5.** Models and results from machine learning classifiers. While the result of random forest classifier showed similar importance of predictors, other classifier models produced a better fit with *θ*_W_ than *F*100kb. Abbreviations: *θ*_W_ = Watterson’s Theta, *F*100kb = *F*_ROH >100kb_, NA = not applicable.

| Model algorithm | Model predictor | Scikit-learn code  of tuned model | Model accuracy | Importance of  *θ*_W_ | Importance of  *F*100kb |
| --- | --- | --- | --- | --- | --- |
| Random Forest | *θ*_W_, *F*100kb | RandomForestClassifier(  bootstrap=False,  max_depth=10,  min_samples_split=1,  n_estimators=200,  random_state=seed) | 0.42 | 0.438 | 0.562 |
| K-Nearest Neighbors | *θ*_W_ | KNeighborsClassifier(  n_neighbors=17) | 0.63 | NA | NA |
| K-Nearest Neighbors | *F*100kb | KNeighborsClassifier(  n_neighbors=6) | 0.46 | NA | NA |
| Linear  Support Vector Machine | *θ*_W_, *F*100kb | SVC(  C=0.1,  degree=1,  kernel='linear') | 0.58 | -0.475  (coefficient) | 0.075  (coefficient) |
| Non-linear  Support Vector Machine | *θ*_W_ | SVC(  C=1,  degree=1,  gamma='auto',  kernel='sigmoid') | 0.58 | NA | NA |
| Non-linear  Support Vector Machine | *F*100kb | SVC(  C=100,  degree=1) | 0.42 | NA | NA |

Table S6 (separate file). Results of applying the proposed genetic criterion with the effective population sizes based on the median population size from the Red List or estimated using ‘*currentNe*’. Information on mutation rates and generation times were obtained from previous literature and the COMBINE database. The original IUCN categories were shown side-by-side with the genetic categories for comparison. The column “genetic_category_beforeHcutoff_10%Nc” represents genetic categories after the *H*_T_:*H*_O_ cutoff and *N*_e_ cutoff based on 10% *N*_e_/*N*_c_ ratio but before applying the relative *H*_O_ value cutoff. The column “genetic_category_beforeHcutoff_100%Nc” represents genetic categories after applying the *H*_T_:*H*_O_ cutoff and *N*_e_ cutoff based on 100% *N*_e_/*N*_c_ ratio but before applying the relative *H*_O_ value cutoff. The column “genetic_category_final_10%Nc” represents genetic categories after applying the *H*_T_:*H*_O_ cutoff and *N*_e_ cutoff based on 10% *N*_e_/*N*_c_ ratio and the relative *H*_O_ value cutoff. The column “genetic_category_final_100%Nc” represents genetic categories after applying the *H*_T_:*H*_O_ cutoff and *N*_e_ cutoff based on 100% *N*_e_/*N*_c_ ratio and the relative *H*_O_ value cutoff. When a species category was changed due to the relative *H*_O_ value cutoff, it was indicated with yellow color (for the genetic criterion based on 10% *N*_e_/*N*_c_ ratio) or orange color (for the genetic criterion based on 100% *N*_e_/*N*_c_ ratio). The species’ heterozygosity values whose genetic category was changed after applying the relative *H*_O_ value cutoff are italicized. The maximum heterozygosity values of genetically Threatened species (i.e., species of VU, EN, or CR before applying the relative *H*_O_ value cutoff) within each taxonomic order are bolded. The genetically Threatened species with the maximum heterozygosity value could be different between 10% *N*_e_/*N*_c_ ratio or 100% *N*_e_/*N*_c_ ratio used, so we filled the cell with yellow (for the case of 10% *N*_e_/*N*_c_ ratio) or orange (for the case of 100% *N*_e_/*N*_c_ ratio).

Table S7 (separate file). NCBI accession numbers for reference genomes and NCBI BioProject numbers for resequencing data are listed along with information on the specific population of each species (geographic locality).

Dataset S1 (separate file). Information on the species analyzed in this study. The species information (*N* = 82) includes taxonomic information (species names according to different data sources, taxonomic Order, and taxonomic Family), the Red List information (category, population trend, and geographic range), eco-evolutionary factors (trophic level, habitat type, habitat breadth, and body mass; note that habitat type is not used in the statistical analyses due to its biased distribution), estimated values of GD metrics (nucleotide diversity, Watterson’s Theta, Tajima’s *D*, heterozygosity per population, heterozygosity per individual, *F*100kb = *F*_ROH >100kb_, *F*1Mb = *F*_ROH >1Mb_), and genomic statistics (Reference genome - assembly level, contig N50, scaffold N50; resequencing data - sample size, sequencing chemistry, mean sequencing depth per individual, average of depth per species, and standard deviation of depth per species).

Dataset S2 (separate file). The “IUCN” dataset used for the PGLS between IUCN categories and GD metrics. This dataset includes all the species having their own Red List assessment, excluding “Data-Deficient” species (*N* = 72); Dataset S2 is a subset of Dataset S1.

Dataset S3 (separate file). The “EcoEvo” dataset used for the PGLS between eco-evolutionary factors and GD metrics. This dataset includes all the species having their own eco-evolutionary factors in the COMBINE database (*N* = 63); Dataset S3 is a subset of Dataset S1.

**SI References**

1. Lê, S., Josse, J. & Husson, F. FactoMineR: An R Package for Multivariate Analysis. *J Stat Softw* **25**, 1–18 (2008).

2. Mundry, R. Statistical issues and assumptions of phylogenetic generalized least squares. in *Modern phylogenetic comparative methods and their application in evolutionary biology: concepts and practice* (ed. Gabriel, L.) 131–153 (Springer, 2014).

3. Bromham, L., Rambaut, A. & Harvey, P. H. Determinants of rate variation in mammalian DNA sequence evolution. *J Mol Evol* **43**, 610–621 (1996).

4. Nevo, E. & Beiles, A. Genetic diversity and ecological heterogeneity in amphibian evolution. *Copeia* 565–592 (1991).

5. Brüniche-Olsen, A., Kellner, K. F., Belant, J. L. & DeWoody, J. A. Life-history traits and habitat availability shape genomic diversity in birds: implications for conservation. *Proc R Soc B* **288**, 20211441 (2021).

6. Soria, C. D., Pacifici, M., Di Marco, M., Stephen, S. M. & Rondinini, C. COMBINE: a coalesced mammal database of intrinsic and extrinsic traits. *Ecology* **102**, e03344 (2021).

7. Martin, A. P. & Palumbi, S. R. Body size, metabolic rate, generation time, and the molecular clock. *Proc Natl Acad Sci* **90**, 4087–4091 (1993).

8. Lino, A., Fonseca, C., Rojas, D., Fischer, E. & Ramos Pereira, M. J. A meta-analysis of the effects of habitat loss and fragmentation on genetic diversity in mammals. *Mamm Biol* **94**, 69–76 (2019).
